# Prevention of Hepatitis B Virus Infection in the United States: Recommendations of the Advisory Committee on Immunization Practices

**DOI:** 10.15585/mmwr.rr6701a1

**Published:** 2018-01-12

**Authors:** Sarah Schillie, Claudia Vellozzi, Arthur Reingold, Aaron Harris, Penina Haber, John W. Ward, Noele P. Nelson

**Affiliations:** 1 Division of Viral Hepatitis, National Center for HIV/AIDS, Viral Hepatitis, STD, and TB Prevention, CDC; 2 University of California, Berkeley School of Public Health, Berkeley, California; 3 Division of Healthcare Quality Promotion, National Center for Emerging and Zoonotic Infectious Diseases, CDC

## Abstract

Hepatitis B virus (HBV) is transmitted via blood or sexual contact. Persons with chronic HBV infection are at increased risk for cirrhosis and liver cancer and require medical care. This report updates and summarizes previously published recommendations from the Advisory Committee on Immunization Practices (ACIP) and CDC regarding the prevention of HBV infection in the United States. ACIP recommends testing all pregnant women for hepatitis B surface antigen (HBsAg), and testing HBsAg-positive pregnant women for hepatitis B virus deoxyribonucleic acid (HBV DNA); administration of HepB vaccine and hepatitis B immune globulin (HBIG) for infants born to HBV-infected women within 12 hours of birth, followed by completion of the vaccine series and postvaccination serologic testing; universal hepatitis B vaccination within 24 hours of birth, followed by completion of the vaccine series; and vaccination of children and adolescents aged <19 years who have not been vaccinated previously. ACIP recommends vaccination of adults at risk for HBV infection, including universal vaccination of adults in settings in which a high proportion have risk factors for HBV infection and vaccination of adults requesting protection from HBV without acknowledgment of a specific risk factor. These recommendations also provide CDC guidance for postexposure prophylaxis following occupational and other exposures. This report also briefly summarizes previously published American Association for the Study of Liver Diseasest guidelines for maternal antiviral therapy to reduce perinatal HBV transmission

## Introduction

Hepatitis B virus (HBV) is transmitted through percutaneous (i.e., puncture through the skin) or mucosal (i.e., direct contact with mucous membranes) exposure to infectious blood or body fluids. HBV is highly infectious, can be transmitted in the absence of visible blood (*1*,*2*), and remains viable on environmental surfaces for at least seven days (*3*). Persons with chronic infection (e.g., those with persistent hepatitis B surface antigen [HBsAg] in the serum for at least 6 months following acute infection) serve as the main reservoir for HBV transmission (*4*).

This report summarizes and consolidates previously published recommendations from the Advisory Committee on Immunization Practices (ACIP) and CDC. It also contains updates to recommendations for the prevention of HBV infection in the United States. A list of frequently used abbreviations is provided (Box 1).

BOX 1 **Abbreviations used in this report**

AASLD American Association for the Study of Liver Diseases

ACIP Advisory Committee on Immunization Practices

anti-HBc antibody to hepatitis B core antigen

anti-HBe antibody to hepatitis B e antigen

anti-HBs antibody to hepatitis B surface antigen

HBeAg hepatitis B e antigen

HBIG hepatitis B immune globulin

HBsAg hepatitis B surface antigen

HBV hepatitis B virus

HBV DNA hepatitis B virus deoxyribonucleic acid

HCP health care personnel

HCV hepatitis C virus

HepB hepatitis B

HIV human immunodeficiency virus

IDSA Infectious Diseases Society of America

IDU Injection-drug use

IgM Immunoglobulin class M

IgG Immunoglobulin class G

MSM men who have sex with men

NNDSS National Notifiable Diseases Surveillance System

PHBPP Perinatal Hepatitis B Prevention Program

PWID persons who inject drugs

QALY quality-adjusted life-year

STI sexually transmitted infection

VAERS Vaccine Adverse Events Reporting System

VSD Vaccine Safety Datalink

## New or Updated Recommendations

The following recommendations are new or updated:

- universal hepatitis B (HepB) vaccination within 24 hours of birth for medically stable infants weighing ≥2,000 grams;
- testing HBsAg-positive pregnant women for hepatitis B virus deoxyribonucleic acid (HBV DNA);
- postvaccination serologic testing for infants whose mother’s HBsAg status remains unknown indefinitely (e.g., when a parent or person with lawful custody surrenders an infant confidentially shortly after birth);
- single-dose revaccination for infants born to HBsAg-positive women not responding to the initial vaccine series;
- vaccination for persons with chronic liver disease (including, but not limited to, those with hepatitis C virus [HCV] infection, cirrhosis, fatty liver disease, alcoholic liver disease, autoimmune hepatitis, and an alanine aminotransferase [ALT] or aspartate aminotransferase [AST] level greater than twice the upper limit of normal); and
- removal of permissive language for delaying the birth dose until after hospital discharge.

This report also briefly summarizes American Association for the Study of Liver Diseases (AASLD) guidelines for maternal antiviral therapy to reduce perinatal HBV transmission, published previously (*5*). Recommendations from the Infectious Diseases Society of America (IDSA) regarding vaccination of the immunocompromised host are published separately (*6*).

## Methods

ACIP’s Hepatitis Work Group comprises professionals from academic medicine (pediatrics, family medicine, internal medicine, infectious disease, occupational health, and preventive medicine specialists), federal and state public health agencies, and medical societies.* The Work Group reviewed epidemiology and literature, directed an economic analysis, and deliberated upon recommendations. The Work Group considered existing published ACIP and CDC vaccine recommendations in summarizing recommendations contained herein for the prevention of HBV infection.

This report updates and supplants ACIP recommendations for HepB vaccination of children and adults published previously (*7*,*8*). This report incorporates ACIP and CDC recommendations published previously (*9*–*11*).

Guidelines from AASLD inform the use of antiviral therapy among pregnant women with elevated HBV DNA for the purpose of preventing perinatal HBV transmission. Surveillance data were obtained from the National Notifiable Diseases Surveillance System (NNDSS) (https://wwwn.cdc.gov/nndss/).

Data informing clarifications to the recommendations were summarized on the basis of findings from literature searches that were completed on May 11, 2016. Two search terms were used to ascertain data regarding maximum number of doses for dialysis patients and minimum intervals for dialysis dosing: “Hepatitis b vacc* dialysis boost*” and “Dialysis hepatitis b vacc* schedule.” Epidemiologic and vaccine coverage data were reviewed, as well as publicly available data on the number of infant abandonments and safely surrendered infants. The literature searches included clinical trials and comparative studies conducted worldwide and published in English since 2000. All studies yielding pertinent information were eligible for inclusion. Search results were supplemented by additional relevant papers identified by subject matter experts on the Work Group. Per the ACIP process, it was predetermined that Grading of Recommendations Assessment, Development and Evaluation (GRADE) was not required for these updates of existing recommendations.

To assess vaccine safety, the Work Group searched two postlicensure surveillance systems for adverse events from 2005 through 2015: the Vaccine Adverse Events Reporting System (VAERS) (https://vaers.hhs.gov) and the Vaccine Safety Datalink (VSD) (https://www.cdc.gov/vaccinesafety/ensuringsafety/monitoring/vsd). VAERS is a national passive surveillance system, and VSD conducts population-based vaccine safety studies. VAERS can generate vaccine safety hypotheses but cannot assess causality and is subject to several limitations, including reporting biases and inconsistent data quality (*12*,*13*). VSD can be used to assess hypotheses that arise from reviews of medical literature, reports to VAERS, changes in immunization schedules, or the introduction of new vaccines (*14*).

During February–September 2016, the Work Group held five teleconference meetings. Work Group and ACIP members also reviewed and commented on a draft of the statement prior to the ACIP’s October 2016 meeting. A summary of Work Group discussions was presented to ACIP on October 19, 2016. At that time, ACIP members voted to approve a draft HepB vaccine recommendations statement, including recommending universal HepB vaccination within 24 hours of birth for medically stable infants weighing ≥2,000 grams. In January 2017, the Work Group held a teleconference meeting to review results of an economic analysis of single-dose revaccination for infants born to HBsAg-positive women. Results from that analysis were presented to ACIP on February 22, 2017. Recommendations were not evaluated using GRADE, but expert opinion was used to shape the recommendations. At that time, ACIP members voted to approve language for single-dose revaccination for infants (regardless of birth weight) born to HBsAg-positive women. Modifications were made to the ACIP statement during the subsequent review process at CDC to update and clarify wording in the report.

## HBV Background

### Epidemiology

**FIGURE 1:**
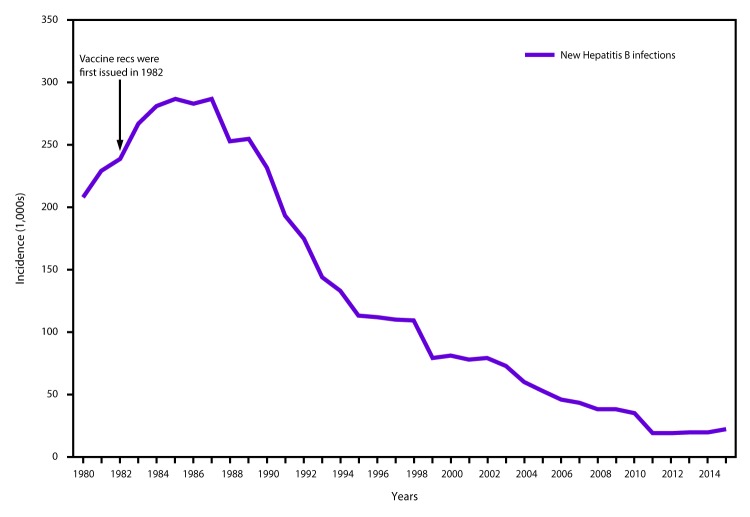
Incidence of hepatitis B virus infection — National Notifiable Diseases Surveillance System, United States, 1980–2015

In 2015, a total of 3,370 cases of acute HBV infection were reported to CDC. The actual number of acute cases is believed to be 6.5 times the number of reported cases in any year. It is estimated that 21,900 new cases of HBV occurred in 2015 after under-ascertainment and under-reporting were considered (*4*). The rate of reported acute HBV infections declined 88.5% since recommendations for HepB vaccination were first issued, from 9.6 cases per 100,000 population in 1982 to 1.1 cases per 100,000 population in 2015 (*15*), although the rate of acute HBV infections remained fairly stable during 2010–2015 (*4*) (Figure 1). The 2015 incidence is greatest for persons aged 30–39 years (2.6 per 100,000 population). In 2015, persons aged ≤19 years had the lowest incidence (0.02 cases per 100,000 population), likely a result of routine infant vaccination. Although the incidence of acute HBV infection is greater for males than for females, the gap has narrowed; in 2015, the rate for males was approximately 1.6 times higher than that for females (1.3 cases and 0.8 cases per 100,000 population, respectively) (*4*). During 2009–2013, the combined incidence of acute HBV infection in three states (Kentucky, Tennessee, and West Virginia) increased 114% and was associated with increasing injection-drug use (*16*).

On the basis of national health survey data, it is estimated that approximately 850,000 persons are living with HBV infection (prevalence) in the United States (*17*,*18*). Studies based on data from countries of persons migrating to the United States and census data indicate that the total prevalence of chronic hepatitis B might be as high as 2.2 million persons (*19*), suggesting that the national health survey-based estimate might be conservative. Foreign-born persons account for approximately 95% of newly reported chronic infections in the United States (*20*); the prevalence of chronic HBV infection is approximately 3.5% among foreign-born persons (*19*), and the majority of chronic HBV infections in the United States are among Asians/Pacific Islanders.

### Strategy to Eliminate HBV

In 1991, the United States adopted a strategy for universal HepB vaccination of infants (*21*). A comprehensive strategy to eliminate HBV transmission evolved over the ensuing 3 decades and encompasses 1) routine testing of all pregnant women for HBsAg and prophylaxis for infants born to HBsAg-positive mothers, 2) universal vaccination of infants beginning at birth, 3) routine vaccination of previously unvaccinated children and adolescents, and 4) vaccination of adults at risk for HBV infection (*7*–*11*,*21*–*26*). Preventing perinatal transmission relies upon testing all pregnant women for HBsAg and administering timely prophylaxis (HepB vaccine and hepatitis B immune globulin [HBIG]) to infants born to infected mothers. Universal HepB vaccination of all infants beginning at birth provides a critical safeguard and prevents infection among infants born to HBsAg-positive mothers not identified prenatally (e.g., in situations where the mother was not tested or when testing, interpretation, or transcription errors occurred). Vaccination of children and adolescents not previously vaccinated and vaccination of adults at risk for HBV infection (e.g., by sexual or percutaneous exposure and international travelers to certain countries) is recommended to prevent HBV transmission outside of the perinatal setting (Box 2).

BOX 2 Strategy to eliminate HBV transmission in the United States*

- Screening of all pregnant women for HBsAg
  - HBV DNA testing for HBsAg-positive pregnant women, with suggestion of maternal antiviral therapy to reduce perinatal transmission when HBV DNA is >200,000 IU/mL
  - Prophylaxis (HepB vaccine and HBIG) for infants born to HBsAg-positive^†^ women
- Universal vaccination of all infants beginning at birth^§,¶^ as a safeguard for infants born to HBV-infected mothers not identified prenatally
- Routine vaccination of previously unvaccinated children aged <19 years
- Vaccination of adults at risk for HBV infection, including those requesting protection from HBV without acknowledgment of a specific risk factor

*Sources: Mast EE, Margolis HS, Fiore AE, et al. A comprehensive immunization strategy to eliminate transmission of hepatitis B virus infection in the United States: recommendations of the Advisory Committee on Immunization Practices (ACIP). Part 1: immunization of infants, children, and adolescents. MMWR Recomm Rep 2005;54(No. RR-16):1–31; Mast EE, Weinbaum CM, Fiore AE, et al. A comprehensive immunization strategy to eliminate transmission of hepatitis B virus infection in the United States: recommendations of the Advisory Committee on Immunization Practices (ACIP). Part II: immunization of adults. MMWR Recomm Rep 2006;55(No. RR-16):1–33.

^†^Refer to Table 3 for prophylaxis recommendations for infants born to women with unknown HBsAg status.

^§^Within 24 hours of birth for medically stable infants weighing ≥2,000 grams.

^¶^Refer to Table 3 for birth dose recommendations for infants weighing <2,000 grams.

HBV prevention strategies have been implemented successfully in the United States, but challenges remain. Approximately 88% of commercially insured women and 84% of Medicaid-enrolled women are tested for HBsAg during pregnancy (*27*). In one study of a large health system in northern California, 93% of HBsAg-positive pregnant women were tested for HBV DNA (*28*). Most (94.9%) infants born to infected women receive recommended prophylaxis within 12 hours of birth (*29*). Universal HepB vaccine birth dose coverage, defined as 1 dose of vaccine administered by 3 days of life, is 71.1% (*30*), an increase from 50.1% during 2003–2005 prior to revised ACIP recommendations for the birth dose before hospital discharge (*31*), but below the *Healthy People 2020* target of 85% (*32*). HepB vaccine coverage (≥3 doses) among children aged 19–35 months and 13–17 years is 90.5% (*30*) and 91.4% (*33*), respectively. Vaccine coverage (≥3 doses) is lower among adults: 27.4% among adults who report chronic liver conditions; 31.6% among adults who traveled outside the United States to countries other than Europe, Japan, Australia, New Zealand, or Canada since 1995; and 24.4% among adults with diabetes aged 19–59 years and 12.6% of adults with diabetes aged ≥60 years (*34*). Among health care personnel (HCP), ≥3-dose coverage was 64.7%, an increase from 51% in 1992 shortly after implementation of the Needlestick Safety and Prevention Act (*35*), but well below the *Healthy People 2020* target of 90% (*32*,*34*).

New strategies for further reducing HBV transmission in this report include testing HBsAg-positive pregnant women for HBV DNA to identify infants at greatest risk for infection and guide the use of maternal antiviral therapy (*36*,*37*). Published evidence indicates that maternal antiviral therapy during pregnancy further reduces perinatal HBV transmission; hence, AASLD suggests antiviral therapy when maternal HBV DNA is >200,000 IU/mL (*5*,*38*,*39*).

### Virus Description and Transmission

HBV is a 40–42-nm enveloped virus classified in the *Hepadnaviridae* family. HBV contains a circular, partially double-stranded DNA genome that is 3.2 kb in length. After a susceptible person is exposed, the virus enters the liver via the bloodstream. The liver is the primary site of HBV replication (*40*–*43*).

HBV has been classified by two separate systems: serologic subtype and genotype. Nine serologic subtypes initially were described based on the heterogeneity of HBsAg: adrq+, adrq–, ayr, ayw1, ayw2, ayw3, ayw4, adw2, and adw4 (*44*,*45*). Ten HBV genotypes, designated A–J, have been described. HBV serotypes and genotypes vary geographically. Infection or immunization with one genotype generally confers immunity to all genotypes (*7*,*44*,*46*,*47*).

HBV is highly infectious, can be transmitted in the absence of visible blood (*22*), and remains infectious on environmental surfaces for at least 7 days (*2*,*3*). All HBsAg-positive persons are infectious, but those with elevated HBV DNA or those with hepatitis B e antigen (HBeAg), a protein from the hepatitis B virus that circulates in the blood and is a marker of infectivity, are most infectious. Persons with occult HBV infection (i.e., those who test negative for HBsAg but have detectable HBV DNA) also might transmit infection (*48*).

HBV is transmitted through percutaneous, mucosal, or nonintact skin exposure to infectious blood or body fluids. HBV is concentrated most highly in blood, and percutaneous exposure is an efficient mode of transmission. Semen and vaginal secretions are infectious, and HBV also can be detected in saliva, tears, and bile. Cerebrospinal fluid, synovial fluid, pleural fluid, peritoneal fluid, pericardial fluid, and amniotic fluid are also considered potentially infectious. Urine, feces, vomitus, nasopharyngeal washings, sputum, and sweat are not efficient vehicles of transmission unless they contain blood because they contain low quantities of infectious HBV. HBsAg found in breast milk is also unlikely to lead to transmission, and hence HBV infection is not a contraindication to breastfeeding (*2*,*7*,*22*).

Among adults, HBV is transmitted primarily by percutaneous exposure to blood (e.g., by injection-drug use) and sexual contact. HBV is transmitted efficiently by sexual contact both among heterosexuals and among men who have sex with men (MSM). Risk factors for sexual transmission among heterosexuals include having unprotected sex with an infected partner, having unprotected sex with more than one partner, and a history of another sexually transmitted infection (STI). Risk factors associated with sexual transmission among MSM include having multiple sex partners, history of another STI, and anal intercourse. Transmission can occur from interpersonal contact (e.g., sharing a toothbrush or razor, contact with exudates from dermatologic lesions, or contact with HBsAg-contaminated surfaces) and in settings such as schools, child care centers, and facilities for developmentally disabled persons. Transmission of HBV from transfusion of blood or blood products is rare because of donor screening and viral inactivation procedures. Other possible sources of infection include contaminated medical or dental instruments, unsafe injections, needle-stick injuries, organ transplantation, and dialysis (*49*).

### Clinical Features and Natural History

Clinical manifestations of HBV infection range from asymptomatic infection to fulminant hepatitis. The average incubation period is 60 days (range: 40–90 days) from exposure to onset of abnormal serum ALT levels and 90 days (range: 60–150 days) from exposure to onset of jaundice (*8*,*42*,*43*). Infants, children aged <5 years, and immunosuppressed adults with newly acquired HBV infection typically are asymptomatic, whereas symptomatic illness is noted in 30%–50% of older children, adolescents, and adults (*7*,*8*,*44*,*50*). When present, signs and symptoms include nausea, vomiting, abdominal pain, fever, dark urine, changes in stool color, hepatomegaly, splenomegaly, and jaundice. Malaise and anorexia might precede jaundice by 1–2 weeks. Fulminant HBV infection is uncommon (<1%) but often results in death or liver failure necessitating liver transplantation. Extrahepatic manifestations of disease (e.g., skin rash, arthralgias, and arthritis) also might occur (*51*). The fatality rate among persons with reported cases of acute HBV infection is <1.5%, with the highest rates in adults aged ≥55 years. Because a substantial number of infections are asymptomatic and therefore are not reported, the overall fatality rate among all persons with HBV infection is likely lower (*8*).

Chronic infection occurs among 80%–90% of persons infected during infancy, 30% of persons infected before age 6 years, and <1%–12% of persons infected as an older child or adult (*7*,*52*–*54*). Approximately 95% of primary infections in immunocompetent adults are self-limited, with elimination of the virus from blood and generally immunity to reinfection. Chronic infection develops more frequently in immunosuppressed persons (e.g., hemodialysis patients and persons with human immunodeficiency virus [HIV] infection) (*54*,*55*) and persons with diabetes (*54*). Chronic HBV infection can result in cirrhosis of the liver, liver cancer, liver failure, and death. Approximately 25% of persons who become chronically infected during childhood and 15% of those who become chronically infected after childhood will die prematurely from cirrhosis or liver cancer (*8*,*56*–*58*).

There are four phases of chronic HBV infection: immune tolerant, immune active, immune inactive, and reactivation. Chronically infected persons do not necessarily pass through these phases in a linear fashion. Persons in the immune tolerant phase have no or minimal hepatic inflammation or fibrosis; most chronically infected children will remain in the immune tolerant phase until late childhood or adolescence. The immune active phase is characterized by an active immune response resulting in hepatic inflammation, with or without fibrosis. Persons who remain in the immune active phase for prolonged periods of time are at high risk for developing cirrhosis and hepatocellular carcinoma. Persons in the immune inactive phase have improvement of hepatic inflammation and fibrosis. Risk for progression to hepatocellular carcinoma is lower among persons in the immune inactive phase compared with the active phase. Persons in the reactivation phase have active liver inflammation with or without fibrosis (*44*,*59*–*61*). HBV reactivation might occur with immunosuppressive therapy or treatment for HCV (*62*).

No specific treatment exists for acute HBV infection; supportive care is the mainstay of therapy. Guidelines for management of chronic HBV infection in children and adults, including disease monitoring and antiviral therapy, are available (*5*). Antiviral therapy generally should be initiated in patients with chronic HBV infection who are likely to respond to treatment and who are at high risk for liver-related morbidity (*5*). Maternal antiviral therapy to reduce perinatal transmission is suggested for HBsAg-positive pregnant women whose HBV DNA level is >200,000 IU/mL (*5*).

In areas in which HBV is highly endemic, HBV frequently is transmitted perinatally from HBV-infected pregnant women to their newborns. The majority of cases of perinatal HBV transmission occur during delivery, with rare instances of in utero transmission (*63*). HBV transmission might occur in germ cell lines, as the virus has been detected in sperm, oocytes, and embryos. Available data do not support the need for a cesarean delivery among HBV-infected pregnant women with low HBV DNA (*63*). Prior to the widespread availability of postexposure prophylaxis, the proportion of infants born to HBsAg-positive women acquiring HBV infection was approximately 30% for those born to HBeAg-negative mothers and 85% for those born to HBeAg-positive mothers. With postexposure prophylaxis, comprised of HepB vaccine and HBIG at birth, followed by completion of the HepB vaccine series, 0.7%–1.1% of infants develop infection (*28*,*29*,*64*); infants born to mothers with high viral loads are at greatest risk for infection despite receipt of HepB vaccine and HBIG (*29*). Unvaccinated infants and children are also at risk for horizontal transmission from infected household and other contacts.

### Interpretation of Serologic Markers

Serologic markers for HBV infection include HBsAg, antibody to HBsAg (anti-HBs), immunoglobulin class M (IgM) antibodies to hepatitis B core antigen (IgM anti-HBc), and immunoglobulin class G (IgG) anti-HBc (IgG anti-HBc) (*49*,*65*,*66*). At least one serologic marker is present during the different phases of infection. HBV DNA is a measure of viral load and reflects viral replication (*49*) (Table 1). Hepatitis B e antigen (HBeAg) can be detected in persons with acute or chronic HBV infection; the presence of HBeAg correlates with viral replication and high infectivity; antibody to HBeAg (anti-HBe) correlates with the loss of replicating virus, although reversion to HBeAg positivity can occur (*7*).

**TABLE 1:** Typical interpretation of test results for hepatitis B virus infection

HBsAg	Total anti-HBc	IgM anti-HBc	Anti-HBs	HBV DNA	Interpretation
-	-	-	-	-	Never infected
+	-	-	-	+ or -	Early acute infection; transient (up to 18 days) after vaccination
+	+	+	-	+	Acute infection
-	+	+	+ or -	+ or -	Acute resolving infection
-	+	-	+	-	Recovered from past infection and immune
+	+	-	-	+	Chronic infection
-	+	-	-	+ or -	False-positive (i.e., susceptible); past infection; “low-level” chronic infection; or passive transfer of anti-HBc to infant born to HBsAg-positive mother
-	-	-	+	-	Immune if anti-HBs concentration is ≥10 mIU/mL after vaccine series completion; passive transfer after hepatitis B immune globulin administration

**Abbreviations:** - = negative; + = positive; anti-HBc = antibody to hepatitis B core antigen; anti-HBs = antibody to hepatitis B surface antigen; HBsAg = hepatitis B surface antigen; HBV DNA = hepatitis B virus deoxyribonucleic acid; IgM = immunoglobulin class M.

A confirmed positive HBsAg result indicates current HBV infection, either acute or chronic. All HBsAg-positive persons are infectious. If HBsAg persists for >6 months, spontaneous clearance is unlikely, and the infection is deemed chronic. HBV DNA can be detected prior to the detection of HBsAg in an infected person. Occult infection occurs when HBsAg is undetectable despite the presence of HBV DNA (*66*–*68*). Transient HBsAg positivity can occur up to 18 days following vaccination (up to 52 days among hemodialysis patients) and is clinically insignificant (*69*).

**FIGURE 2:**
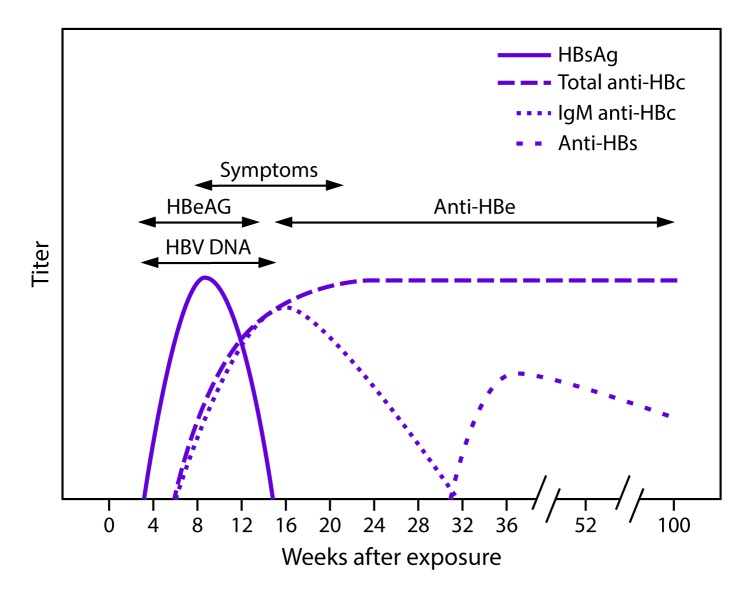
Acute hepatitis B virus infection with recovery **Abbreviations: **anti-HBc = antibody to hepatitis B core antigen; anti-HBe = antibody to hepatitis B e antigen; anti-HBs = antibody to hepatitis B surface antigen; HBeAg = hepatitis B e antigen; HBsAg = hepatitis B surface antigen; HBV DNA = hepatitis B virus deoxyribonucleic acid; IgM = immunoglobulin class M.

In acute HBV infection, anti-HBc (initially both IgM and IgG) appears 1–2 weeks after the appearance of HBsAg (*49*) (Figure 2). IgM anti-HBc often becomes undetectable within 6 months, and IgG anti-HBc predominates and remains detectable for a lengthy period of time, often life-long (*65*,*66*). The presence of IgM anti-HBc is indicative of acute infection, while IgG anti-HBc indicates past infection (*65*,*66*). In persons who recover from HBV infection, HBsAg is eliminated from the blood and anti-HBs develops, typically within 3–4 months. The presence of anti-HBs is generally indicative of immunity to HBV infection (*8*). Anti-HBs also can be detected for 4–6 months following HBIG administration (*10*). Persons who recover from natural HBV infection are typically positive for both anti-HBs and anti-HBc, whereas persons who respond to HepB vaccine are positive only for anti-HBs. Approximately 0.5%–2% of persons with chronic infection spontaneously clear HBsAg yearly; anti-HBs will develop in the majority of these persons (*8*).

In certain persons, anti-HBc is the only serologic marker detected. Isolated anti-HBc-positivity can be detected following HBV infection in persons who have recovered but whose anti-HBs levels have waned; in populations with a high prevalence of HBV infection, isolated anti-HBc likely indicates previous infection with loss of anti-HBs. Some chronically infected persons with isolated anti-HBc-positivity have circulating HBsAg that is not detectable by a laboratory assay. HBV DNA has been detected in <10% of persons with isolated anti-HBc (*70*,*71*), although the presence of detectable HBV DNA might fluctuate (*72*). These persons are unlikely to transmit infection except under circumstances in which they are the source of a large exposure, such as a blood transfusion (*8*,*73*). Persons who are HBsAg-negative and anti-HBc-positive can experience reactivation of infection during chemotherapy or immunosuppressive therapy, with reappearance of HBsAg (*49*). Infection with a mutant HBV strain can result in positive laboratory tests for HBsAg, total anti-HBc, anti-HBs, and HBV DNA, with a negative IgM anti-HBc.

Perinatal HBV infection in a child aged ≤24 months is typically asymptomatic although fulminant hepatitis can occur; a positive HBsAg test, positive HBeAg test, or detectable HBV DNA may be considered laboratory evidence of perinatal HBV in an infant born to an HBV-infected mother if timing criteria are met (*74*). Infants who are born to HBsAg-positive mothers and who do not become infected might have detectable anti-HBc for up to 24 months after birth from passively acquired maternal antibody (*7*).

### Adults at Risk for HBV Infection

In 2015, CDC received 3,370 surveillance case-reports of acute HBV infection. Of 2,207 case-reports with risk information, 1,151 (52.2%) indicated no risk for HBV during the 6 weeks to 6 months prior to illness onset, and the remainder indicated at least one risk factor. Injection-drug use and multiple sex partners were the most common reported sources of HBV transmission (*4*).

**Injection-drug use.** Injection-drug use was reported by 30.3% of 1,657 new reported HBV cases that included information about injection-drug use (*4*). Since 2009, there has been an increase in acute HBV infection among non-Hispanic whites aged 30–39 years residing in nonurban areas reporting injection-drug use as a risk factor (*16*). Chronic HBV infection has been identified in 3.5%-20.0% (midpoint estimate: 11.8%) of persons who inject drugs (PWID) in a variety of settings (*75*) and 22.6% of PWID have evidence of past infection (*75*). The proportion of HBV cases reporting injection-drug use in three states (Kentucky, Tennessee, and West Virginia) increased significantly, from 53% during 2006–2009 to 75% during 2010–2013 (p<0.001, chi-square) (*16*).

**Sexual (heterosexual and MSM) exposure.** Among persons with case-reports of HBV infection with information about sexual exposure, 26.4% reported having two or more sexual partners, 3.3% reported sexual contact with an HBV-infected person, and 11.8% of males reported having had sex with another male (*4*). As many as 10%–40% of adults seeking treatment in STI clinics have evidence of current or past HBV infection. Among adults with acute HBV infection, 39% were screened or sought care for an STI prior to becoming infected with HBV (*76*).

**Household contacts.** An estimated 45% of persons living in households with others with chronic HBV infection have serologic evidence of past HBV infection, and 16% have evidence of current infection (CDC, unpublished data, 2017. Prior to universal infant vaccination, the risk for infection was greatest among unvaccinated children living with a person with chronic HBV infection in a household or in an extended family setting (*67*,*77*,*78*).

**Developmentally disabled persons in long-term-care facilities.** Developmentally disabled persons in residential and nonresidential facilities historically have had a chronic HBV infection prevalence as high as 20%. The prevalence of infection has declined substantially since the implementation of routine HepB vaccination in these settings (*79*–*82*).

**Correctional facilities.** The prevalence of chronic HBV infection has been higher among prison inmates (1.0%–3.7%) than among the general population (*83*,*84*), reflecting an overrepresentation of persons entering correctional facilities with risks for HBV infection (e.g., injection-drug use and histories of multiple sex partners).

**Persons at risk for occupational exposure to HBV.** Before HepB vaccination was widely implemented, HBV infection was recognized as a common occupational risk among HCP (*85*,*86*). Routine HepB vaccination of HCP and the use of standard precautions have resulted in a 98% decline in HBV infections from 1983 through 2010 among HCP (*10*). The Occupational Safety and Health Administration mandates that employers offer HepB vaccination to all employees who have occupational risk and that postexposure prophylaxis be available following an exposure (*10*,*87*).

**Hemodialysis patients.** Since the initiation of HepB vaccination and additional infection control precautions for hepatitis B in dialysis centers, the incidence of HBV infection among hemodialysis patients has declined approximately 95% (*88*,*89*). Since 1995, the annual incidence has been stable and HBsAg seroprevalence has remained at 1% (*90*). Receipt of dialysis was reported in <1% of acute HBV surveillance cases with information reported to CDC (*4*).

**Persons with HCV infection.** The number of reported HCV cases in four Appalachian states (Kentucky, Tennessee, Virginia, and West Virginia) increased 364% during 2006–2012 among persons aged ≤30 years, with injection-drug use as the most common reported risk factor (*91*). The increase in HCV infections occurred concomitantly with an increase in HBV infections among young adults in rural communities in Appalachian states.

**Persons with chronic liver disease.** Persons with chronic liver disease (e.g., cirrhosis, fatty liver disease, alcoholic liver disease, and autoimmune hepatitis) are not at increased risk for HBV infection unless they have percutaneous or mucosal exposure to blood or body fluids. However, concurrent chronic HBV infection might increase the risk for progressive chronic liver disease in these persons (*92*).

**Travelers to countries where HBV is endemic.** Short-term travelers to countries in which HBV infection is of high or intermediate endemicity (Box 3) typically are at risk for infection only through exposure to blood in medical or disaster-relief activities, receipt of medical care that involves parenteral exposures, sexual activity, or drug use. Monthly incidence of 25–420 per 100,000 travelers has been reported among long-term travelers to countries where the disease is endemic (*93*).

BOX 3 Prevalence of chronic hepatitis B virus infection, by country*

**High (≥8% prevalence): **Angola, Benin, Burkina Faso, Burundi, Cameroon, Central African Republic, Congo, Côte d’Ivoire, Djibouti, Equatorial Guinea, Gabon, Gambia, Ghana, Guinea, Haiti, Kiribati, Kyrgyzstan, Laos, Liberia, Malawi, Mali, Mauritania, Mongolia, Mozambique, Namibia, Nauru, Niger, Nigeria, Niue, Papua New Guinea, Senegal, Sierra Leone, Solomon Islands, Somalia, South Sudan, Sudan, Swaziland, Togo, Tonga, Uganda, Vanuatu, Vietnam, Yemen, and Zimbabwe.

**Intermediate (5%–7.9% prevalence): **Albania, Bhutan, Cape Verde, China, Democratic Republic of the Congo, Ethiopia, Kazakhstan, Kenya, Marshall Islands, Moldova, Oman, Romania, Rwanda, Samoa, South Africa, Tajikistan, Tanzania, Thailand, Tunisia, Tuvalu, Uzbekistan, and Zambia.

**Low Intermediate (2%–4.9% prevalence): **Algeria, Azerbaijan, Bangladesh, Belarus, Belize, Brunei Darussalam, Bulgaria, Cambodia, Colombia, Cyprus, Dominican Republic, Ecuador, Eritrea, Federated States of Micronesia, Fiji, Georgia, Italy, Jamaica, Kosovo, Libya, Madagascar, Myanmar, New Zealand, Pakistan, Palau, Philippines, Peru, Russia, Saudi Arabia, Singapore, South Korea, Sri Lanka, Suriname, Syria, Tahiti, and Turkey.

**Low (≤1.9% prevalence): **Afghanistan, Argentina, Australia, Austria, Bahrain, Barbados, Belgium, Bolivia, Bosnia and Herzegovina, Brazil, Canada, Chile, Costa Rica, Croatia, Cuba, Czech Republic, Denmark, Egypt, France, Germany, Greece, Guatemala, Hungary, Iceland, India, Indonesia, Iran, Iraq, Ireland, Israel, Japan, Jordan, Kuwait, Lebanon, Lithuania, Malaysia, Mexico, Morocco, Nepal, Netherlands, Nicaragua, Norway, Palestine, Panama, Poland, Portugal, Qatar, Serbia, Seychelles, Slovakia, Slovenia, Spain, Sweden, Switzerland, Ukraine, UK, United Arab Emirates, United States of America, and Venezuela.

**No data: **Andorra, Antigua and Barbuda, Armenia, The Bahamas, Botswana, Chad, Comoros, Cook Islands, Dominica, El Salvador, Finland, Grenada, Guinea- Bissau, Guyana, Honduras, Latvia, Lesotho, Lithuania, Luxembourg, Macedonia, Maldives, Malta, Mauritius, Monaco, Montenegro, North Korea, Paraguay, Saint Kitts and Nevis, Saint Lucia, Saint Vincent and the Grenadines, San Marino, Sao Tome and Principe, Timor-Leste, Trinidad and Tobago, Turkmenistan, and Uruguay.

* **Source:** CDC. Travelers health: infectious diseases related to travel. Atlanta, GA: US Department of Health and Human Services, CDC; 2017.

**Persons with HIV.** Approximately 10% of HIV-positive persons are coinfected with HBV (*94*–*97*). Chronic HBV infection has been identified in 6%–14% of HIV-positive persons, including in 9%–17% of MSM and in 7%–10% of PWID (*98*). Coinfected persons have increased rates of cirrhosis and liver-related mortality (*99*).

**Persons with diabetes.** Compared with adults without diabetes, adults with diabetes have a 60% higher prevalence of past or present HBV infection and twice the odds of acquiring acute HBV. Repeated outbreaks of HBV infection associated with assisted blood glucose monitoring underscore the continued risk for this population (*100*–*102*). Data also suggest the possibility of a higher case-fatality proportion among persons with diabetes acutely infected with HBV compared with those without diabetes (*9*).

## Prophylaxis Against HBV Infection

### Hepatitis B Vaccines and Hepatitis B Immune Globulins

HepB vaccination is the mainstay of HBV prevention efforts; HBIG is generally used as an adjunct to HepB vaccine in infants born to HBsAg-positive mothers and in certain other postexposure prophylaxis situations. The first HepB vaccines consisted of plasma-derived HBsAg. Recombinant HepB vaccines containing yeast-derived HBsAg purified by biochemical and biophysical separation techniques replaced the plasma-derived vaccines in the United States by the late 1980s (*64*,*103*,*104*). HepB vaccines recommended for use in the United States are formulated to contain 10–40 *µ*g of HBsAg protein/mL and do not contain thimerosal as a preservative (*105*). HBIG can augment protection until a response to vaccination is attained. For those who do not respond to HepB vaccination, HBIG administered alone is the primary means of protection after an HBV exposure. HBIG provides passively acquired anti-HBs and temporary protection (i.e., 3–6 months). Passively acquired anti-HBs can be detected for 4–6 months after administration of HBIG (*10*).

HepB vaccines are available as a single-antigen formulation and in combination with other vaccines. The two single-antigen vaccines recommended for use in the United States, Engerix-B (GlaxoSmithKline Biologicals, Rixensart, Belgium) and Recombivax HB (Merck & Co., Inc., Whitehouse Station, New Jersey), are used for the vaccination of persons starting at birth. Of the two combination vaccines, Pediarix (GlaxoSmithKline Biologicals, Rixensart, Belgium) is used for the vaccination of persons aged 6 weeks–6 years and contains recombinant HBsAg, diphtheria and tetanus toxoids and acellular pertussis adsorbed, and inactivated poliovirus and Twinrix (GlaxoSmithKline Biologicals, Rixensart, Belgium) is used for the vaccination of persons aged ≥18 years and contains recombinant HBsAg and inactivated hepatitis A virus (Table 2). Comvax (Merck & Co., Inc., Whitehouse Station, New Jersey), which was used previously for the vaccination of persons aged 6 weeks–15 months and contained recombinant HBsAg and *Haemophilus b* conjugate vaccine, has not been available for purchase directly from Merck since January 1, 2015. Discontinuation of Comvax was not related to any product safety or manufacturing issues. Aluminum salts generally are used as adjuvants to enhance the immune response of vaccinated persons.

**TABLE 2:** Recommended doses of hepatitis B vaccine, by group and vaccine type

Age group (yrs)	Single-antigen vaccine						Combination vaccine			
	Recombivax		Engerix			Pediarix*			Twinrix^†^	
	Dose (*µ*g)	Vol (mL)		Dose (*µ*g)	Vol (mL)	Dose (*µ*g)		Vol (mL)	Dose (*µ*g)	Vol (mL)
Birth–10	5	0.5		10	0.5	10*		0.5	N/A	N/A
11–15	10^§^	1		N/A	N/A	N/A		N/A	N/A	N/A
11–19	5	0.5		10	0.5	N/A		N/A	N/A	N/A
≥20	10	1		20	1	N/A		N/A	20^†^	1
Hemodialysis patients and other immune-compromised persons										
<20	5	0.5		10	0.5	N/A		N/A	N/A	N/A
≥20	40	1		40	2	N/A		N/A	N/A	N/A

**Abbreviation:** N/A = not applicable.

* Pediarix is approved for use in persons aged 6 weeks through 6 years (prior to the 7th birthday).

^†^ Twinrix is approved for use in persons aged ≥18 years.

^§^ Adult formulation administered on a 2-dose schedule.

Two HBIG products are licensed for use in the United States: HepaGam B (Cangene Corporation, Winnipeg, Canada) and Nabi-HB (Biotest Pharmaceuticals Corporation, Boca Raton, Florida). HBIG is prepared from the plasma of donors with high concentrations of anti-HBs. Source plasma tests negative for evidence of HIV, HBV, and HCV. Investigational nucleic acid testing for hepatitis A virus and parvovirus B19 also is performed on pooled samples of source plasma. The manufacturing process contains two steps to inactivate viruses in the final product: the solvent and detergent step inactivates enveloped viruses, and the virus filtration step removes viruses based on their size. HBIG products licensed for use in the United States contain no preservative and are intended for single use only (*106*).

### Vaccine-Induced Seroprotection

The presence of anti-HBs typically indicates immunity against HBV infection. Immunocompetent children and adults who have vaccine-induced anti-HBs levels of ≥10 mIU/mL 1–2 months after having received a complete HepB vaccine series are considered seroprotected and deemed vaccine responders (*107*). Vaccine-induced seroprotection is considered a surrogate of clinical protection. Anti-HBs levels wane over time following vaccination related in part to the age at vaccination. Approximately 16% of persons vaccinated at age <1 year have antibody levels of ≥10 mIU/mL 18 years following vaccination, compared with 74% for those vaccinated at age ≥1 year (*10*). However, persons initially responding to the full 3-dose HepB vaccine series and who are later found to have anti-HBs <10 mIU/mL remain protected. Most persons (88%) who receive a challenge dose of HepB vaccine 30 years after HepB vaccination as children or adults develop an antibody response of ≥10 mIU/mL indicating persistent immunity to HBV infection (*108*). Data from this and other studies suggests protection against acute symptomatic and chronic HBV infection persists for 30 years or more among immunocompetent persons who originally responded to HepB vaccine (*108*–*110*).

The 3-dose HepB vaccine series produces a protective antibody response (anti-HBs ≥10 mIU/mL) in approximately 95% of healthy infants overall (response is lower for infants with lower birth weights) (*64*) and >90% of healthy adults aged <40 years (*111*,*112*). Among healthy infants, 25% and 63% achieve anti-HBs levels ≥10 mIU/mL after the first and second dose, respectively. Among healthy adults aged <40 years, 30%–55% and 75% achieve anti-HBs levels ≥10 mIU/mL after the first and second dose, respectively (*7*,*8*,*64*). Vaccine response is decreased among infants weighing <2000 grams and older adults. Other factors (e.g., smoking, obesity, aging, chronic medical conditions, drug use, diabetes, male sex, genetic factors, and immune suppression) contribute to a decreased response to vaccine (*113*–*116*). Although immunogenicity is lower among immunocompromised persons, those who achieve and maintain seroprotective antibody levels before exposure to HBV have a high level of protection (*8*).

**Birth dose.** A birth dose of HepB vaccine serves as postexposure prophylaxis to prevent perinatal HBV infection among infants born to HBV-infected mothers. Although infants requiring postexposure prophylaxis should be identified by maternal HBsAg testing, administration of a birth dose to all infants (even without HBIG) serves as a safeguard to prevent perinatal transmission among infants born to HBsAg-positive mothers not identified prenatally because of lack of maternal HBsAg testing or failures in reporting test results. HepB vaccine or HBIG given alone are 75% and 71% effective in preventing perinatal HBV transmission, respectively; their combined efficacy is 94% (*29*,*52*,*117*). The birth dose also provides protection to infants at risk from household exposure after the perinatal period (*29*,*64*).

Vaccination produces seroprotection in 98% of healthy term infants. Vaccine response is lower among infants with birth weights <2000 grams (*64*). A study among low birth weight infants demonstrated that more infants achieved seroprotective anti-HBs levels when vaccine was initiated at 1 month of age versus within the first 3 days of life (96% vs. 68%, p<0.02) (*118*). Vaccine response among infants does not vary appreciably by maternal HBsAg status or HBIG administration (*64*).

**Adolescents.** Approximately 95% of adolescents achieve seroprotection following HepB vaccination with a complete series (*7*). The adult (10 *µ*g) dose of Recombivax HB administered using a 2-dose compressed schedule at 0 and 4 months or 0 and 6 months for persons aged 11–15 years produces seroprotection proportions nearly equivalent to those obtained with the standard regimen of 5 *µ*g administered on a 3-dose schedule at 0, 1, and 6 months (99.2% vs. 98.3%) (*119*,*120*). Data on long-term antibody persistence or protection among adolescents for 2-dose schedules are lacking.

**Adults.** Vaccination with a complete series results in seroprotection in >90% of healthy adults aged <40 years. Response decreases with age, and seroprotection is achieved in 75% of persons aged 60 years (*8*).

**Diabetes.** A review of studies assessing HepB vaccine response among persons with diabetes mellitus demonstrated seroprotection in 93.9% for children with diabetes mellitus compared with 100% for children without diabetes mellitus (*112*,*121*).

Among adults, 88.2% of those with diabetes mellitus, compared with 93.6% of those without diabetes mellitus, achieved seroprotection (*112*). Among hemodialysis/chronic kidney disease patients, the median proportion protected was 60.1% for those with diabetes mellitus, compared with 75.1% for those without diabetes mellitus (*112*).

**Immunocompromising conditions.** The humoral response to HepB vaccine is reduced in children and adults who are immunocompromised (e.g., hematopoietic stem cell transplant recipients, patients undergoing chemotherapy, and HIV-infected persons) (*122*,*123*). Modified dosing regimens, including a doubling of the standard antigen dose or administration of additional doses, might increase response rates. However, data on response to these alternative vaccination schedules are limited (*6*).

### Vaccine Safety

In prelicensure trials, adverse events following HepB vaccination were most commonly injection site reactions and mild systemic reactions (*106*). Commonly reported mild adverse events from postmarketing data include pain (3%–29%), erythema (3%), swelling (3%), fever (1%–6%), and headache (3%) (*124*). The estimated incidence of anaphylaxis among HepB vaccine recipients is 1.1 per million vaccine doses (*125*). In 2011, the Institute of Medicine concluded that the evidence convincingly supports a causal relationship between HepB vaccine and anaphylaxis in yeast-sensitive persons, and that the evidence is inadequate to accept or reject a causal relation between HepB vaccine and several neurologic, chronic, and autoimmune diseases (*126*).

During early postlicensure surveillance, several adverse events following HepB vaccination have been described in the scientific literature, including Guillain-Barré Syndrome (GBS), chronic fatigue syndrome, optic neuritis, multiple sclerosis, and diabetes mellitus; however, multiple studies have demonstrated no association between receipt of HepB vaccine and these conditions (*126*–*129*). In addition, no evidence of a causal association between rheumatoid arthritis (*130*), Bell’s palsy (*131*), autoimmune thyroid disease (*132*), hemolytic anemia in children (*133*), anaphylaxis (*134*), optic neuritis (*135*), Guillain-Barré Syndrome (*136*), sudden-onset sensorineural hearing loss (*137*), or other chronic illnesses and receipt of HepB vaccine has been demonstrated through analysis of VSD data.

During 2005–2015, a total of 20,231 reports of adverse events following HepB vaccination among all ages were submitted to VAERS. The majority of primary U.S. reports (15,787 of 20,231, 78%) were following HepB vaccine administered with other vaccines on the same visit. Among these, the percentage classified as serious (i.e., if one or more of the following is reported: death, life-threatening illness, hospitalization or prolongation of existing hospitalization, or permanent disability)^†^ was 16.7%, including 402 deaths, of which 388 were among infants aged 6 weeks–23 months (*138*). The most frequently reported adverse events for vaccines given in combination were fever (23%), injection site erythema (11%), and vomiting (10%) (*138*). Among the 4,444 single-antigen HepB reports, 6.5% were classified as serious, including 43 deaths, of which 27 were among infants aged ≤4 weeks. The most frequently reported adverse events for single-antigen HepB vaccine were nausea/dizziness (8%) and fever/headache (7%).

### Vaccination Schedules

Vaccine schedules are determined on the basis of immunogenicity data, and, for infants and children, the need to integrate HepB vaccine into a harmonized immunization schedule (Tables 3 and 4). Primary vaccination generally consists of three intramuscular doses administered on a 0-, 1-, and 6-month schedule (Table 4). Recombivax HB may be administered in a 2-dose schedule at 0 and 4–6 months for persons aged 11–15 years using the adult formulation. Pediarix is administered at ages 2, 4, and 6 months; it is not used for the birth dose. Twinrix may be administered before travel or any other potential exposure on an accelerated schedule at 0, 7, and 21–30 days, followed by a dose at 12 months. HepB vaccination of adult hemodialysis patients consists of high-dose (40 *µ*g) Recombivax HB administered on a 0-, 1-, and 6-month schedule or high-dose (40 *µ*g) Engerix-B administered on a 0-, 1-, 2-, and 6-month schedule (*106*).

**TABLE 3:** Hepatitis B vaccine schedules for infants, by infant birthweight and maternal HBsAg status

Birthweight	Maternal HBsAg status	Single-antigen vaccine		Single-antigen + combination vaccine	
		Dose	Age	Dose	Age
≥2,000 g	Positive	1	Birth (≤12 hrs)	1	Birth (≤12 hrs)
		HBIG^§^	Birth (≤12 hrs)	HBIG	Birth (≤12 hrs)
		2	1–2 mos	2	2 mos
		3	6 mos^¶^	3	4 mos
				4	6 mos^¶^
	Unknown*	1	Birth (≤12 hrs)	1	Birth (≤12 hrs)
		2	1–2 mos	2	2 mos
		3	6 mos^¶^	3	4 mos
				4	6 mos^¶^
	Negative	1	Birth (≤24 hrs)	1	Birth (≤24 hrs)
		2	1–2 mos	2	2 mos
		3	6–18 mos^¶^	3	4 mos
				4	6 mos^¶^
<2,000 g	Positive	1	Birth (≤12 hrs)	1	Birth (≤12 hrs)
		HBIG	Birth (≤12 hrs)	HBIG	Birth (≤12 hrs)
		2	1 mos	2	2 mos
		3	2–3 mos	3	4 mos
		4	6 mos^¶^	4	6 mos^¶^
	Unknown	1	Birth (≤12 hrs)	1	Birth (≤12 hrs)
		HBIG	Birth (≤12 hrs)	HBIG	Birth (≤12 hrs)
		2	1 mos	2	2 mos
		3	2–3 mos	3	4 mos
		4	6 mos^¶^	4	6 mos^¶^
	Negative	1	Hospital discharge or age 1 mo	1	Hospital discharge or age 1 mo
		2	2 mos	2	2 mos
		3	6–18 mos^¶^	3	4 mos
				4	6 mos^¶^

**Abbreviations:** HBIG = hepatitis B immune globulin; HBsAg = hepatitis B surface antigen.

* Mothers should have blood drawn and tested for HBsAg as soon as possible after admission for delivery; if the mother is found to be HBsAg positive, the infant should receive HBIG as soon as possible but no later than age 7 days.

^†^ Pediarix should not be administered before age 6 weeks.

^§^ HBIG should be administered at a separate anatomical site from vaccine.

^¶^ The final dose in the vaccine series should not be administered before age 24 weeks (164 days).

**TABLE 4:** Hepatitis B vaccine schedules for children, adolescents, and adults

Age group	Schedule* (interval represents time in months from first dose)
Children (1–10 yrs)	0, 1, and 6 mos
	0, 1, 2, and 12 mos
Adolescents (11–19 yrs)	0, 1, and 6 mos
	0, 12, and 24 mos
	0 and 4–6 mos^†^
	0, 1, 2, and 12 mos 0, 7 days, 21–30 days, 12 mos^§^
Adults (≥20 yrs)	0, 1, and 6 mos
	0, 1, 2, and 12 mos 0, 1, 2, and 6 mos^¶^ 0, 7 days, 21–30 days, 12 mos^§^

* Refer to package inserts for further information. For all ages, when the HepB vaccine schedule is interrupted, the vaccine series does not need to be restarted. If the series is interrupted after the first dose, the second dose should be administered as soon as possible, and the second and third doses should be separated by an interval of at least 8 weeks. If only the third dose has been delayed, it should be administered as soon as possible. The final dose of vaccine must be administered at least 8 weeks after the second dose and should follow the first dose by at least 16 weeks; the minimum interval between the first and second doses is 4 weeks. Inadequate doses of hepatitis B vaccine or doses received after a shorter-than-recommended dosing interval should be readministered, using the correct dosage or schedule. Vaccine doses administered ≤4 days before the minimum interval or age are considered valid. Because of the unique accelerated schedule for Twinrix, the 4-day guideline does not apply to the first three doses of this vaccine when administered on a 0-day, 7-day, 21–30-day, and 12-month schedule (new recommendation).

^†^A 2-dose schedule of Recombivax adult formulation (10 µg) is licensed for adolescents aged 11–15 years. When scheduled to receive the second dose, adolescents aged >15 years should be switched to a 3-dose series, with doses 2 and 3 consisting of the pediatric formulation administered on an appropriate schedule.

^§^ Twinrix is approved for use in persons aged ≥18 years and is available on an accelerated schedule with doses administered at 0, 7, 21–30 days, and 12 months.

^¶^ A 4-dose schedule of Engerix administered in two 1 mL doses (40 µg) on a 0-, 1-, 2-, and 6-month schedule is recommended for adult hemodialysis patients.

Alternative vaccination schedules (e.g., 0, 1, and 4 months or 0, 2, and 4 months) have been demonstrated to elicit dose-specific and final rates of seroprotection similar to those obtained on a 0-, 1-, and 6-month schedule. Increasing the interval between the first 2 doses has little effect on immunogenicity or the final antibody concentration (*139*–*141*). The third dose confers the maximum level of seroprotection and provides long-term protection (*142*). Longer intervals between the last 2 doses (e.g., 11 months) result in higher final antibody levels (*142*) but might increase the risk for acquisition of HBV infection among persons who have a delayed response to vaccination. Higher geometric mean titers are associated with longer persistence of measurable anti-HBs.

### Response to Revaccination

A challenge dose of HepB vaccine may be used to determine the presence of vaccine-induced immunologic memory through generation of an anamnestic response. The term “booster dose” has been used to refer to a dose of HepB vaccine administered after a primary vaccination series to provide rapid protective immunity against significant infection (i.e., infection resulting in serologic test results positive for HBV and/or clinically significant disease). Among persons who were vaccinated prior to age 1 year and found to have anti-HBs levels <10 mIU/mL 6–18 years later, a single challenge dose of HepB vaccine resulted in anti-HBs levels ≥10 mIU/mL in 60%–97% of those tested. Similar results were found among persons initially vaccinated at age ≥1 year (*10*). Immunocompetent persons with a response ≥10 mIU/mL following a challenge dose are considered protected, regardless of subsequent declines in anti-HBs (*10*,*109*).

One study found that of infants born to HBsAg-positive women who were not infected at birth and who did not respond to a primary vaccine series, all developed seroprotective levels of anti-HBs after receipt of 3 additional doses (*143*). No data exist that suggest that children who have no detectable antibody after 6 doses of vaccine benefit from additional doses.

### Maternal Antiviral Therapy for Preventing Perinatal HBV Transmission

Antiviral therapy (i.e., lamivudine, telbivudine, and tenofovir) has been studied as an intervention to reduce perinatal HBV transmission among pregnant women with high HBV DNA levels (e.g., average HBV DNA levels of 7.6 log10 IU/mL) (*144*). Maternal antiviral therapy started at 28–32 weeks’ gestation, as an adjunct to HepB vaccine and HBIG administered to the infant shortly after delivery, has been associated with significantly reduced rates of perinatal HBV transmission (*5*). The use of lamivudine and telbivudine is limited by viral resistance and mutations. Tenofovir is not associated with resistance and is the preferred agent (*5*). Available data support the safety of tenofovir during pregnancy, although its use might be associated with reduced bone mineral content in infants with in utero exposure (*5*,*39*,*63*,*144*–*146*). AASLD suggests antiviral therapy to reduce perinatal HBV transmission when maternal HBV DNA is >200,000 IU/mL. Maternal therapy is generally discontinued at birth to 3 months postpartum (*5*).

### Cost-Effectiveness Considerations

HBV prevention strategies targeting perinatal transmission are considered very cost-effective (i.e., an incremental cost-effectiveness ratio <$25,000). The current strategy of administering HepB vaccine and HBIG within 12 hours of birth for infants born to HBsAg-positive mothers and universal infant vaccination prior to hospital discharge has an incremental cost-effectiveness ratio of $6,957 per quality-adjusted life year (QALY) saved when compared with a strategy of universal infant HepB vaccination prior to hospital discharge alone (*147*). CDC’s U.S. Perinatal Hepatitis B Prevention Program (https://www.cdc.gov/hepatitis/partners/perihepbcoord.htm), which provides case management services to infants born to HBsAg-positive women, also has been demonstrated to decrease infections, increase QALYs saved, and be a cost-effective use of resources (*148*). A strategy of testing HBsAg-positive pregnant women for HBV DNA, followed by maternal antiviral prophylaxis for women with high HBV DNA, would cost an additional $3 million but would save 2,080 QALYs and prevent 324 chronic HBV infections, and therefore would be considered cost-effective, with an incremental cost-effectiveness ratio of $1,583 per QALY saved (*36*).

Cost-effectiveness also has been assessed for HBV prevention strategies outside of the perinatal setting. Vaccinating adults aged 20–59 years with diabetes mellitus costs $75,094 per QALY saved; cost-effectiveness ratios increase with age at vaccination (*149*). Among previously vaccinated current HCP (including those in training), pre-exposure anti-HBs testing followed by revaccination and retesting (if necessary, based on anti-HBs levels), compared with no intervention, was not considered cost-effective with an incremental cost per QALY saved of $3–$4 million at year one and approximately $800,000 over 10 years (*150*).

## Recommendations

This section contains guidance for the prevention of HBV infection, including ACIP recommendations for HepB vaccination of infants, children, adolescents, and adults (Box 4) and CDC and ACIP recommendations for HBV prophylaxis following occupational and nonoccupational exposures, respectively.

BOX 4 **Persons recommended to receive hepatitis B vaccination**

• All infants

• Unvaccinated children aged <19 years

• Persons at risk for infection by sexual exposure

- Sex partners of hepatitis B surface antigen (HBsAg)–positive persons
- Sexually active persons who are not in a long-term, mutually monogamous relationship (e.g., persons with more than one sex partner during the previous 6 months)
- Persons seeking evaluation or treatment for a sexually transmitted infection
- Men who have sex with men
  - Persons at risk for infection by percutaneous or mucosal exposure to blood
- Current or recent injection-drug users
- Household contacts of HBsAg-positive persons
- Residents and staff of facilities for developmentally disabled persons
- Health care and public safety personnel with reasonably anticipated risk for exposure to blood or blood-contaminated body fluids
- Hemodialysis patients and predialysis, peritoneal dialysis, and home dialysis patients
- Persons with diabetes aged 19–59 years; persons with diabetes aged ≥60 years at the discretion of the treating clinician
  - Others
- International travelers to countries with high or intermediate levels of endemic hepatitis B virus (HBV) infection (HBsAg prevalence of ≥2%)
- Persons with hepatitis C virus infection
- Persons with chronic liver disease (including, but not limited to, persons with cirrhosis, fatty liver disease, alcoholic liver disease, autoimmune hepatitis, and an alanine aminotransferase [ALT] or aspartate aminotransferase [AST] level greater than twice the upper limit of normal)
- Persons with HIV infection
- Incarcerated persons
- All other persons seeking protection from HBV infection

### Prevention of Perinatal HBV Transmission

#### Identification and Management of HBV-Infected Pregnant Women

- All pregnant women should be tested for HBsAg during an early prenatal visit (e.g., first trimester) in each pregnancy, even if they have been vaccinated or tested previously. Testing those pregnant women known to be chronically infected with HBV provides documentation of the positive HBsAg test result obtained during pregnancy and helps to ensure that their infants will be identified for timely prophylaxis.
  - All HBsAg-positive pregnant women should be tested for HBV DNA to guide the use of maternal antiviral therapy during pregnancy for the prevention of perinatal HBV transmission (new recommendation).
  - AASLD suggests maternal antiviral therapy when the maternal HBV DNA is >200,000 IU/mL (new recommendation).
  - All HBsAg-positive pregnant women should be referred to their jurisdiction’s Perinatal Hepatitis B Prevention Program (PHBPP) for case management to ensure that their infants receive timely prophylaxis and follow-up. A copy of the original laboratory report indicating the pregnant woman's HBsAg-positive status should be provided to the hospital or birthing facility where the delivery is planned and to the HCP who will care for the newborn infant.
  - All HBsAg-positive pregnant women should receive information concerning HBV that discusses the potential use of antiviral therapy, the importance of prophylaxis for their infant (HepB vaccine and HBIG within 12 hours of birth), completion of the vaccine series, and postvaccination serologic testing.
- Women not tested prenatally, those with clinical hepatitis, and those whose behaviors place them at high risk for HBV infection (e.g., recent or current injection-drug use, having had more than one sex partner in the previous 6 months or an HBsAg-positive sex partner, having been evaluated or treated for a STI) should be tested at the time of admission to the hospital or birthing facility for delivery.
- All laboratories that provide HBsAg testing of pregnant women should use a Food and Drug Administration–licensed or approved HBsAg test and should perform testing according to the manufacturer's labeling, including testing of initially reactive specimens with a licensed neutralizing confirmatory test. When pregnant women are tested for HBsAg at the time of admission for delivery, shortened testing protocols may be used and initially reactive results reported to expedite administration of postexposure prophylaxis of infants. Commercial laboratories should be encouraged to capture pregnancy status for women tested for HBsAg to aid in identification of HBV-infected pregnant women.

#### Management of Infants Born to Women Who Are HBsAg-Positive

- All infants born to HBsAg-positive women should receive HepB vaccine and HBIG within 12 hours of birth, administered at different injection sites (e.g., separate limbs). Only single-antigen HepB vaccine should be used for the birth dose (Table 3).
- Infants born to women for whom HBsAg testing results during pregnancy are not available but other evidence suggestive of maternal HBV infection exists (e.g., presence of HBV DNA, HBeAg-positive, or mother known to be chronically infected with HBV) should be managed as if born to an HBsAg-positive mother (new recommendation).
- The HepB vaccine series should be completed according to the recommended schedule for infants born to HBsAg-positive mothers. The final dose in the series should not be administered before age 24 weeks (164 days). Although not indicated in the manufacturers’ package labeling, Pediarix may be used for infants aged ≥6 weeks born to HBsAg-positive mothers to complete the vaccine series after receipt of a birth dose of single-antigen HepB vaccine and HBIG.
- For infants weighing <2,000 grams, the birth dose (i.e., the initial HepB vaccine dose) should not be counted as part of the vaccine series because of the potentially reduced immunogenicity of HepB vaccine in these infants; 3 additional doses of vaccine (for a total of 4 doses) should be administered beginning when the infant reaches age 1 month. The final dose in the series should not be administered before age 24 weeks (164 days).
- Postvaccination serologic testing for anti-HBs and HBsAg should be performed after completion of the vaccine series at age 9–12 months (generally at the next well-child visit following completion of the HepB vaccine series). Anti-HBs testing should be performed using a method that allows detection of the protective concentration of anti-HBs (≥10 mIU/mL). Testing should not be performed before age nine months to avoid detection of passive anti-HBs from HBIG administered at birth and to maximize the likelihood of detecting late HBV infection. Anti-HBc testing of infants is not recommended because passively acquired maternal anti-HBc might be detected in infants born to HBsAg-positive mothers up to age 24 months.
  - HBsAg-negative infants with anti-HBs levels ≥10 mIU/mL are protected and need no further medical management.
  - HBsAg-negative infants with anti-HBs <10 mIU/mL should be revaccinated with a single dose of HepB vaccine and receive postvaccination serologic testing 1–2 months later (new recommendation). Infants whose anti-HBs remains <10 mIU/mL following single dose revaccination should receive two additional doses of HepB vaccine to complete the second series, followed by postvaccination serologic testing 1–2 months after the final dose.
  - Based on clinical circumstances or family preference, HBsAg-negative infants with anti-HBs <10 mIU/mL may instead be revaccinated with a second, complete 3-dose series, followed by postvaccination serologic testing performed 1–2 months after the final dose of vaccine.
  - Available data do not suggest a benefit from administering additional HepB vaccine doses to infants who have not attained anti-HBs ≥10 mIU/mL following receipt of two complete HepB vaccine series.
  - HBsAg-positive infants should be referred for appropriate follow-up.
- Infants who are born to HBsAg-positive mothers and receive postexposure prophylaxis may be breastfed beginning immediately after birth.
- For infants transferred to a different facility after birth (e.g., hospital with higher level of neonatal care), staff at the transferring and receiving facilities should communicate regarding the infant’s HepB vaccination and HBIG receipt status to ensure prophylaxis is administered in a timely manner (new recommendation).

#### Management of Infants Born to Women with Unknown HBsAg Status

- Infants born to women for whom HBsAg testing results during pregnancy are not available but other evidence suggestive of maternal HBV infection exists (e.g., presence of HBV DNA, HBeAg-positive, or mother known to be chronically infected with HBV) should be managed as if born to an HBsAg-positive mother (new recommendation). The infant should receive both HepB vaccine and HBIG within 12 hours of birth.
- Women admitted for delivery without documentation of HBsAg test results should have blood drawn and tested as soon as possible.
- While maternal HBsAg test results are pending, infants with birth weights ≥2,000 grams born to women with an unknown HBsAg status should receive the first dose of HepB vaccine (without HBIG) within 12 hours of birth. Only single-antigen HepB vaccine should be used for the birth dose (Table 3).
  - If the mother is determined to be HBsAg-positive, the infant should receive HBIG as soon as possible but no later than age seven days, and the vaccine series should be completed according to the recommended schedule for infants born to HBsAg-positive mothers. The final dose in the series should not be administered before age 24 weeks (164 days). If the mother is determined to be HBsAg-negative, the vaccine series should be completed according to the recommended schedule for infants born to HBsAg-negative mothers. The final dose in the series should not be administered before age 24 weeks (164 days).
- Because of the potentially decreased immunogenicity of vaccine in infants weighing <2,000 grams, these infants should receive both single-antigen HepB vaccine and HBIG, administered at different injection sites (e.g., separate limbs), if the mother's HBsAg status cannot be determined within 12 hours of birth. The birth dose of vaccine should not be counted as part of the 3 doses required to complete the vaccine series; 3 additional doses of vaccine (for a total of 4 doses) should be administered according to a recommended schedule on the basis of the mother's HBsAg test result. The final dose in the series should not be administered before age 24 weeks (164 days).
  - If it is not possible to determine the mother’s HBsAg status (e.g., when a parent or person with lawful custody safely surrenders an infant confidentially shortly after birth), the vaccine series should be completed according to a recommended schedule for infants born to HBsAg-positive mothers (new recommendation). The final dose in the series should not be administered before age 24 weeks (164 days). These infants should receive postvaccination serologic testing at age 9–12 months, and revaccination if necessary (new recommendation).
- Anti-HBs testing should be performed using a method that allows detection of the protective concentration of anti-HBs (≥10 mIU/mL). Testing should not be performed before age nine months to avoid detection of passive anti-HBs from HBIG administered at birth and to maximize the likelihood of detecting late HBV infection. Anti-HBc testing of infants is not recommended because passively acquired maternal anti-HBc might be detected in infants born to HBsAg-positive mothers up to age 24 months.
  - HBsAg-negative infants with anti-HBs levels ≥10 mIU/mL are protected and need no further medical management.
  - HBsAg-negative infants with anti-HBs <10 mIU/mL should be revaccinated with a single dose of HepB vaccine and receive postvaccination serologic testing 1–2 months later (new recommendation). Infants whose anti-HBs remains <10 mIU/mL following single dose revaccination should receive two additional doses of HepB vaccine to complete the second series, followed by postvaccination serologic testing 1–2 months after the final dose.
  - Based on clinical circumstances or family preference, HBsAg-negative infants with anti-HBs <10 mIU/mL may instead be revaccinated with a second, complete 3-dose series, followed by postvaccination serologic testing performed 1–2 months after the final dose of vaccine.
  - Available data do not suggest a benefit from administering additional HepB vaccine doses to infants who have not attained anti-HBs ≥10 mIU/mL following receipt of two complete HepB vaccine series.
  - HBsAg-positive infants should be referred for appropriate follow-up.
- Infants born to mothers with unknown HBsAg status may be breastfed beginning immediately after birth.
- For infants transferred to a different facility after birth (e.g., a hospital with a higher level of neonatal care), staff at the transferring and receiving facilities should communicate regarding the infant’s HepB vaccination and HBIG receipt status to ensure prophylaxis is administered in a timely manner (new recommendation).

### Persons Recommended for HepB Vaccination

#### Universal Vaccination of Infants

- All infants should receive the HepB vaccine series as part of the recommended childhood immunization schedule, beginning at birth as a safety net (Box 4; Table 3).
- For all medically stable infants weighing ≥2,000 grams at birth and born to HBsAg-negative mothers, the first dose of vaccine should be administered within 24 hours of birth (new recommendation). Only single-antigen HepB vaccine should be used for the birth dose.
- Infants weighing <2,000 grams and born to HBsAg-negative mothers should have their first vaccine dose delayed to the time of hospital discharge or age 1 month (even if weight is still <2,000 grams). For these infants, a copy of the original laboratory report indicating that the mother was HBsAg negative during this pregnancy should be placed in the infant's medical record. Infants weighing <2,000 grams at birth have a decreased response to HepB vaccine administered before age 1 month (*118*).
- For infants transferred to a different facility after birth (e.g., a hospital with a higher level of neonatal care), staff at the transferring and receiving facilities should communicate regarding the infant’s HepB vaccination and HBIG receipt status to ensure prophylaxis is administered in a timely manner (new recommendation).
- The final dose in the vaccine series should not be administered before age 24 weeks (164 days).
- In populations with currently or previously high rates of childhood HBV infection (e.g., Alaska Natives; Pacific Islanders; and immigrant families from Asia, Africa, and countries with intermediate or high endemic rates of infection), the first dose of HepB vaccine should be administered at birth and the final dose at age 6–12 months.

#### Vaccination of Children and Adolescents

- HepB vaccination is recommended for all unvaccinated children and adolescents aged <19 years (Box 4).
- Children and adolescents who have not previously received HepB vaccine should be vaccinated routinely at any age (i.e., children and adolescents are recommended for catch-up vaccination) (Table 4).

#### Vaccination of Adults

- HepB vaccination is recommended for all unvaccinated adults at risk for HBV infection and for all adults requesting protection from HBV infection. Acknowledgement of a specific risk factor should not be a requirement for vaccination (Box 4).
- Adults recommended to receive HepB vaccine:
  - Persons at risk for infection by sexual exposure (e.g., sex partners of HBsAg-positive persons, sexually active persons who are not in a mutually monogamous relationship [e.g., persons with more than one sex partner during the previous 6 months], persons seeking evaluation or treatment for a sexually transmitted infection, and MSM).
  - Persons with a history of current or recent injection drug use are at increased risk for HBV infection. An increased incidence of HBV incidence among young adults in rural U.S. communities has been associated with an increase in injection drug use.
  - Other persons at risk for infection by percutaneous or mucosal exposure to blood (household contacts of HBsAg-positive persons; residents and staff of facilities for developmentally disabled persons; health care and public safety personnel with reasonably anticipated risk for exposure to blood or blood-contaminated body fluids, hemodialysis patients and predialysis, peritoneal dialysis, and home dialysis patients; persons with diabetes mellitus aged <60 years and persons with diabetes mellitus aged ≥60 years at the discretion of the treating clinician).
  - Others (international travelers to countries with high or intermediate levels [HBsAg prevalence of ≥2%] [Box 3] of endemic HBV infection, persons with HCV infection, persons with chronic liver disease [including, but not limited to, those with cirrhosis, fatty liver disease, alcoholic liver disease, autoimmune hepatitis, and an ALT or AST level greater than twice the upper limit of normal] [new recommendation], persons with HIV infection, incarcerated persons, all other persons seeking protection from HBV infection without acknowledgement of a specific risk factor).

#### Vaccination of Pregnant Women

- Pregnant women who are identified as being at risk for HBV infection during pregnancy (e.g., having more than one sex partner during the previous 6 months, been evaluated or treated for an STI, recent or current injection-drug use, or having had an HBsAg-positive sex partner) should be vaccinated.
- Pregnant women at risk for HBV infection during pregnancy should be counseled concerning other methods to prevent HBV infection.

### Implementation Strategies

#### Delivery Hospital Policies and Procedures

- All delivery hospitals and birthing facilities should implement policies and procedures to ensure identification of infants born to HBsAg-positive mothers and infants born to mothers with unknown HBsAg status, initiation of prophylaxis for these infants, and routine birth dose for medically stable infants weighing ≥2,000 grams within 24 hours of birth. Such policies and procedures should include standing orders and electronic medical record reminders or prompts.

#### Case-Management Programs to Prevent Perinatal HBV Infection

- States and localities should establish case-management programs, including appropriate policies, procedures, laws, and regulations to ensure that all pregnant women are tested for HBsAg during each pregnancy and that those who are HBsAg-positive are tested for HBV DNA to guide maternal antiviral therapy. Infants born to HBsAg-positive women and women with unknown HBsAg status also should receive case management.

#### Settings Providing Services to Adults

- In settings in which a high proportion of persons have risk factors for HBV infection (e.g., health care settings targeting services to injection-drug users, correctional facilities, institutions and nonresidential day care facilities for developmentally disabled persons), all adults should be assumed to be at risk for HBV infection and should be offered HepB vaccination if they have not previously completed vaccination.
- HCP should implement standing orders to administer HepB vaccine as part of routine services to adults who have not completed the vaccine series and make HepB vaccination a standard component of evaluation and treatment for STIs and HIV/AIDS.
- When feasible, HepB vaccination should be offered in outreach and other settings in which services are provided to persons at risk for HBV infection (e.g., needle-exchange programs, HIV testing sites, HIV prevention programs, and homeless shelters).
- In medical settings, HCP should implement standing orders to identify adults recommended for HepB vaccination and administer vaccination as part of routine services.

### Postexposure Prophylaxis

This section provides recommendations for management of persons who are exposed to HBV through a distinct, identifiable exposure to blood or body fluids that contain blood, in occupational and nonoccupational settings.

- Wounds and skin sites that have been in contact with blood or body fluids should be washed with soap and water; mucous membranes should be flushed with water. Using antiseptics (e.g., 2%–4% chlorhexidine) for wound care or expressing fluid by squeezing the wound further have not been shown to reduce the risk for HBV transmission; however, the use of antiseptics is not contraindicated. The application of caustic agents (e.g., bleach) or the injection of antiseptics or disinfectants into the wound is not recommended.

#### Occupational Settings

##### Vaccinated HCP

- For vaccinated HCP (who have written documentation of a complete HepB vaccine series) with subsequent documented anti-HBs ≥10 mIU/mL, testing the source patient for HBsAg is unnecessary. No postexposure prophylaxis for HBV is necessary, regardless of the source patient's HBsAg status (Table 5).

**TABLE 5:** Postexposure management of health care personnel after occupational percutaneous or mucosal exposure to blood or body fluids, by health care personnel HepB vaccination and response status

HCP status	Postexposure testing		Postexposure prophylaxis			Postvaccination serologic testing	
	Source patient (HBsAg)	HCP testing (anti-HBs)	HBIG	Vaccination			
Documented responder after complete series	No action needed						
Documented nonresponder after two complete series	Positive/unknown	–*	HBIG x2 separated by 1 month	–		N/A	
	Negative	No action needed					
Response unknown after complete series	Positive/unknown	<10 mIU/mL	HBIG x1	Initiate revaccination		Yes	
	Negative	<10 mIU/mL	None				
	Any result	≥10 mIU/mL			Initiate revaccination		Yes
			No action needed				
Unvaccinated/incompletely vaccinated or vaccine refusers	Positive/unknown	–	HBIG x1	Complete vaccination		Yes	
	Negative	–	None	Complete vaccination		Yes	

**Abbreviations**: anti HBs = antibody to hepatitis B surface antigen; HBIG = hepatitis B immune globulin; HBsAg = hepatitis B surface antigen; HCP = health care personnel; N/A = not applicable.

* Not indicated.
- For vaccinated HCP (who have written documentation of a complete HepB vaccine series) without previous anti-HBs testing, the HCP should be tested for anti-HBs and the source patient (if known) should be tested for HBsAg as soon as possible after the exposure. Anti-HBs testing should be performed using a method that allows detection of the protective concentration of anti-HBs (≥10 mIU/mL). Testing the source patient and the HCP should occur simultaneously; testing the source patient should not be delayed while waiting for the HCP anti-HBs test results, and likewise, testing the HCP should not be delayed while waiting for the source patient’s HBsAg results (Table 5).
  - If the HCP has anti-HBs <10 mIU/mL and the source patient is HBsAg-positive or has an unknown HBsAg status, the HCP should receive 1 dose of HBIG and be revaccinated as soon as possible after the exposure. HepB vaccine may be administered simultaneously with HBIG at a separate anatomical injection site (e.g., separate limb). The HCP should then receive the second 2 doses of HepB vaccine to complete the second series (likely 6 doses total when accounting for the original series) according to the vaccination schedule. So the HCP’s vaccine response status can be documented for future exposures, anti-HBs testing should be performed 1–2 months after the final vaccine dose.
  - If the HCP has anti-HBs <10 mIU/mL and the source patient is HBsAg-negative, the HCP should receive an additional single HepB vaccine dose, followed by repeat anti-HBs testing 1–2 months later. HCP whose anti-HBs remains <10 mIU/mL should undergo revaccination with two more doses (likely 6 doses total when accounting for the original series). So the HCP’s vaccine response status can be documented for future exposures, anti-HBs testing should be performed 1–2 months after the final dose of vaccine.
  - If the HCP has anti-HBs ≥10 mIU/mL at the time of the exposure, no postexposure HBV management is necessary, regardless of the source patient's HBsAg status.
- For vaccinated HCP with anti-HBs <10 mIU/mL after two complete HepB vaccine series, the source patient should be tested for HBsAg as soon as possible after the exposure. If the source patient is HBsAg-positive or has unknown HBsAg status, the HCP should receive 2 doses of HBIG (*1*,*10*). The first dose should be administered as soon as possible after the exposure, and the second dose should be administered 1 month later. HepB vaccine is not recommended for the exposed HCP who has previously completed two HepB vaccine series. If the source patient is HBsAg-negative, neither HBIG nor HepB vaccine is necessary (Table 5).

##### Unvaccinated HCP

- For unvaccinated or incompletely vaccinated HCP, the source patient should be tested for HBsAg as soon as possible after the exposure. Testing unvaccinated or incompletely vaccinated HCP for anti-HBs is not necessary and is potentially misleading, because anti-HBs ≥10 mIU/mL as a correlate of vaccine-induced protection has only been determined for persons who have completed an approved vaccination series (*107*) (Table 5).
- If the source patient is HBsAg-positive or has an unknown HBsAg status, the HCP should receive 1 dose of HBIG and 1 dose of HepB vaccine administered as soon as possible after the exposure. HepB vaccine may be administered simultaneously with HBIG at a separate anatomical injection site (e.g., separate limb). The HCP should complete the HepB vaccine series according to the vaccination schedule. To document the HCP's vaccine response status for future exposures, anti-HBs testing should be performed approximately 1–2 months after the final vaccine dose. Anti-HBs testing should be performed using a method that allows detection of the protective concentration of anti-HBs (≥10 mIU/mL). Because anti-HBs testing of HCP who received HBIG should be performed after anti-HBs from HBIG is no longer detectable (6 months after administration), it might be necessary to defer anti-HBs testing for a period longer than 1–2 months after the last vaccine dose in these situations (Table 5).
  - HCP with anti-HBs ≥10 mIU/mL after receipt of the primary vaccine series are considered immune. Immunocompetent persons have long-term protection and do not need further periodic testing to assess anti-HBs levels.
  - HCP with anti-HBs <10 mIU/mL after receipt of the primary series should be revaccinated. For these HCP, administration of a second complete series on an appropriate schedule, followed by anti-HBs testing 1–2 months after the final dose, is usually more practical than conducting serologic testing after each additional dose of vaccine. So the HCP’s vaccine response status can be documented for future exposures, anti-HBs testing should be performed 1–2 months after the final vaccine dose.
- If the source patient is HBsAg-negative, the HCP should complete the HepB vaccine series according to the vaccination schedule. So the HCP’s vaccine response status can be documented for future exposures, anti-HBs testing should be performed approximately 1–2 months after the final vaccine dose (Table 5).
  - HCP with anti-HBs ≥10 mIU/mL after receipt of the primary vaccine series are considered immune. Immunocompetent persons have long-term protection and do not need further periodic testing to assess anti-HBs levels.
  - HCP with anti-HBs <10 mIU/mL after receipt of the primary series should be revaccinated. For these HCP, administration of a second complete series on an appropriate schedule, followed by anti-HBs testing 1–2 months after the final dose, is usually more practical than conducting serologic testing after each additional dose of vaccine. So the HCP’s vaccine response status can be documented for future exposures, anti-HBs testing should be performed 1–2 months after the final vaccine dose.

##### Clinical Management of Exposed HCP

- HCP who have anti-HBs <10 mIU/mL (or who are unvaccinated or incompletely vaccinated) and sustain an exposure to a source patient who is HBsAg-positive or has an unknown HBsAg status should undergo baseline testing for HBV infection as soon as possible after the exposure, and follow-up testing approximately 6 months later. Testing immediately after the exposure should consist of total anti-HBc, and follow-up testing approximately 6 months later should consist of HBsAg and total anti-HBc (Table 5).
- HCP exposed to a source patient who is HBsAg-positive or has an unknown HBsAg status do not need to take special precautions to prevent secondary transmission during the follow-up period; however, they should refrain from donating blood, plasma, organs, tissue, or semen (*10*). The exposed HCP does not need to modify sexual practices or refrain from becoming pregnant (*10*). If an exposed HCP is breastfeeding, she does not need to discontinue (*7**,**10*). No modifications to an exposed HCP’s patient-care responsibilities are necessary to prevent transmission to patients based solely on exposure to a source patient who is HBsAg-positive or has an unknown HBsAg status.

##### Previously Vaccinated HCP

**FIGURE 3:**
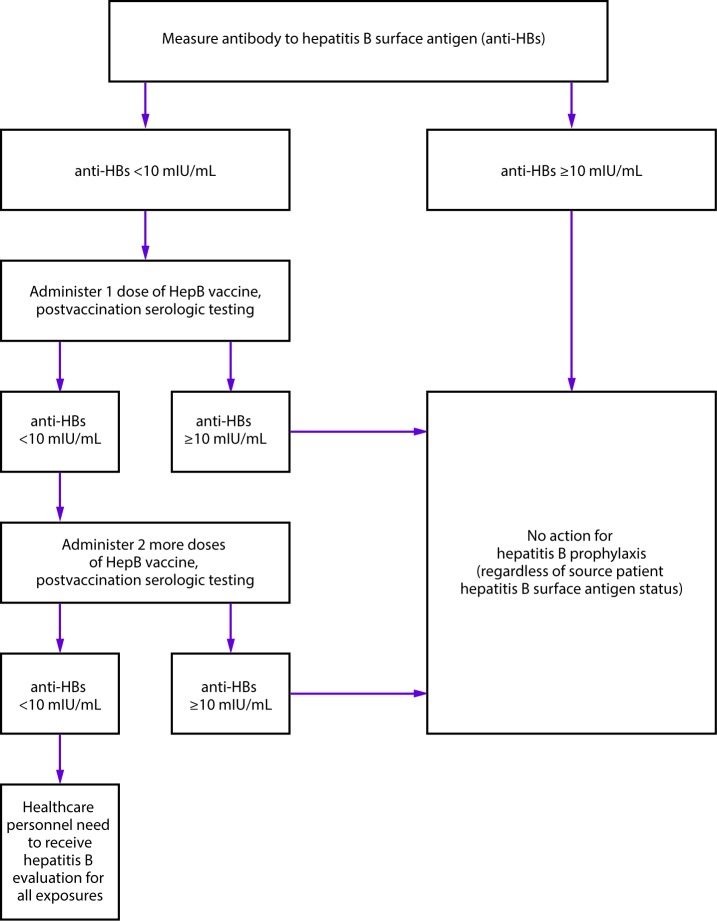
Pre-exposure evaluation for health care personnel previously vaccinated with complete, ≥3-dose HepB vaccine series who have not had postvaccination serologic testing* * Should be performed 1–2 months after the last dose of vaccine using a quantitative method that allows detection of the protective concentration of anti-HBs (≥10 mIU/mL) (e.g., enzyme-linked immunosorbent assay [ELISA]).
  - HCP who cannot provide documentation of 3 doses of HepB vaccine should be considered unvaccinated and should complete the vaccine series. Postvaccination serologic testing for anti-HBs is recommended 1–2 months after the third vaccine dose. HCP who are inadvertently tested before receiving 3 documented doses of HepB vaccine and have anti-HBs ≥10 mIU/mL should not be considered immune because anti-HBs ≥10 mIU/mL is a known correlate of protection only when testing follows a documented 3-dose series. Health care facilities are encouraged to try to locate vaccine records for HCP and to enter all vaccine doses in their state immunization information system.

- Providers should only accept written, dated records as evidence of HepB vaccination (*151*).
- An increasing number of HCP have received routine HepB vaccination during childhood. No postvaccination serologic testing is recommended after routine infant or adolescent HepB vaccination. Because vaccine-induced anti-HBs wanes over time, testing HCP for anti-HBs years after vaccination might not distinguish vaccine nonresponders from responders. Pre-exposure assessment of current or past anti-HBs results upon hire or matriculation, followed by one or more additional doses of HepB vaccine for HCP with anti-HBs <10 mIU/mL and retesting anti-HBs, if necessary, helps to ensure that HCP will be protected if they have an exposure to HBV-containing blood or body fluids (Box 5; Figure 3).
  BOX 5 **Testing anti-HBs for health care personnel (HCP) vaccinated in the past**
  **The issue:** An increasing number of HCP have received routine hepatitis B (HepB) vaccination during childhood. No postvaccination serologic testing is recommended after routine infant or adolescent HepB vaccination. Because vaccine-induced antibody to hepatitis B surface antigen (anti-HBs) wanes over time, testing HCP for anti-HBs years after vaccination might not distinguish vaccine nonresponders from responders.
  **Guidance for health care institutions:** Health care institutions may measure anti-HBs upon hire or matriculation for HCP who have documentation of a complete HepB vaccine series in the past (e.g., as part of routine infant or adolescent vaccination). HCP with anti-HBs <10 mIU/mL should receive one or more additional doses of HepB vaccine and retesting (Figure 3). Institutions that decide to not measure anti-HBs upon hire or matriculation for HCP who have documentation of a complete HepB vaccine series in the past should ensure timely assessment and postexposure prophylaxis following an exposure (Table 5).
  **Considerations:** The risk for occupational HBV infection for vaccinated HCP might be low enough in certain settings so that assessment of anti-HBs status and appropriate follow-up should be done at the time of exposure to potentially infectious blood or body fluids. This approach relies on HCP recognizing and reporting blood and body fluid exposures and therefore may be applied on the basis of documented low risk, implementation, and cost considerations. Certain HCP occupations have lower risk for occupational blood and body fluid exposures (e.g., occupations involving counseling versus performing procedures), and nontrainees have lower risks for blood and body fluid exposures than trainees. Some settings also will have a lower prevalence of HBV infection in the patient population served than in other settings, which will influence the risk for HCP exposure to HBsAg-positive blood and body fluids.

#### Nonoccupational Settings

##### HBsAg-Positive Source

This section provides recommendations for management of persons who are exposed to HBV through a distinct, identifiable exposure to blood or body fluids that contain blood, in nonoccupational settings (Table 6). The exposed person does not need to undergo postvaccination serologic testing following vaccination based solely on being exposed.

**TABLE 6:** Postexposure management after distinct nonoccupational percutaneous or mucosal exposure to blood or body fluids

Exposure*	Management	
	Unvaccinated person	Previously vaccinated person
HBsAg-positive source	HepB vaccine series and HBIG	HepB vaccine dose
HBsAg status unknown for source	Hep B vaccine series	No management

**Abbreviations:** HepB = hepatitis B; HBsAg = hepatitis B surface antigen; HBIG = hepatitis B immune globulin.

* Exposures include percutaneous (e.g., bite or needlestick) or mucosal exposure to blood or body fluids, sex or needle-sharing contact, or victim of sexual assault/abuse.

- Exposed persons who have written documentation of a complete HepB vaccine series and who did not receive postvaccination testing should receive a single dose of HepB vaccine.
- Exposed persons who are in the process of being vaccinated but who have not completed the vaccine series should receive a dose of HBIG and complete the HepB vaccine series (it is not necessary to restart the HepB vaccine series). HepB vaccine may be administered simultaneously with HBIG at a separate anatomical injection site (e.g., separate limb).
- Exposed unvaccinated persons should receive both HBIG and HepB vaccine as soon as possible after exposure (preferably within 24 hours). HepB vaccine may be administered simultaneously with HBIG at a separate anatomical injection site (e.g., separate limbs). Studies are limited on the maximum interval after exposure during which postexposure prophylaxis is effective, but the interval is unlikely to exceed 7 days for percutaneous exposure and 14 days for sexual exposures. The HepB vaccine series should be completed according to the vaccination schedule.

##### HBsAg-Unknown Source

- Exposed persons with written documentation of a complete HepB vaccine series require no further treatment.
- Exposed persons who are in the process of being vaccinated but who are not fully vaccinated should complete the HepB vaccine series (it is not necessary to restart the vaccination series).
- Exposed unvaccinated persons should receive the HepB vaccine series with the first dose administered as soon as possible after exposure, preferably within 24 hours. Studies are limited on the maximum interval after exposure during which postexposure prophylaxis is effective, but the interval is unlikely to exceed 7 days for percutaneous exposure and 14 days for sexual exposures. The vaccine series should be completed according to the vaccination schedule.

### Immunization Management Issues

#### Prevaccination Testing

- Vaccination of persons immune to HBV because of current or previous infection or HepB vaccination does not increase the risk for adverse events (*8*). However, in populations that have high rates of previous HBV infection, prevaccination testing might reduce costs by avoiding vaccination of persons who are already immune. Prevaccination testing consists of testing for HBsAg, anti-HBs, and anti-HBc. Serologic testing should not be a barrier to vaccination of susceptible persons, especially in populations that are difficult to access. Testing is not a requirement for vaccination, and in settings where testing is not feasible, vaccination of recommended persons should continue.
- The first dose of HepB vaccine should typically be administered immediately after collection of the blood for serologic testing. Prevaccination testing is recommended for the following persons (Box 6):
  BOX 6 Persons recommended to receive serologic testing prior to vaccination*
  - Household, sexual, or needle contacts of hepatitis B surface antigen (HBsAg)–positive persons^†^
  - HIV-positive persons^†^
  - Persons with elevated alanine aminotransferase/aspartate aminotransferase of unknown etiology^†^
  - Hemodialysis patients^†^
  - Men who have sex with men^†^
  - Past or current persons who inject drugs^†^
  - Persons born in countries of high and intermediate hepatitis B virus (HBV) endemicity (HBsAg prevalence ≥2%)
  - U.S.-born persons not vaccinated as infants whose parents were born in countries with high HBV endemicity (≥8%)
  - Persons needing immunosuppressive therapy, including chemotherapy, immunosuppression related to organ transplantation, and immunosuppression for rheumatologic or gastroenterologic disorders
  - Donors of blood, plasma, organs, tissues, or semen
  * Serologic testing comprises testing for hepatitis B surface antigen (HBsAg), antibody to HBsAg, and antibody to hepatitis B core antigen.
  ^†^ Denotes persons also recommended for hepatitis B vaccination. Serologic testing should occur prior to vaccination. Serologic testing should not be a barrier to vaccination of susceptible persons. The first dose of vaccine should typically be administered immediately after collection of the blood for serologic testing.
  - household, sexual, or needle-sharing contacts of HBsAg-positive persons;
  - HIV-positive persons;
  - persons with elevated alanine aminotransferase (ALT)/aspartate aminotransferase (AST) of unknown etiology;
  - hemodialysis patients (refer to 2001 CDC recommendations [*88*] for additional information);
  - MSM; and
  - past or current injection-drug users.

#### Testing for HBV Infection

- Testing for HBV infection (consisting of testing for HBsAg, anti-HBs, and anti-HBc) is also recommended for the following persons:
  - persons born in countries of high and intermediate HBV endemicity (HBsAg prevalence ≥2%);
  - U.S.-born persons not vaccinated as infants whose parents were born in countries with high HBV endemicity (≥8%);
  - persons needing immunosuppressive therapy, including chemotherapy, immunosuppression related to organ transplantation, and immunosuppression for rheumatologic or gastroenterologic disorders; and
  - donors of blood, plasma, organs, tissues, or semen.
- All pregnant women should be tested for HBsAg during each pregnancy. Pregnant women with positive HBsAg tests should be tested for HBV DNA.

#### Postvaccination Serologic Testing

- Serologic testing for immunity is not necessary after routine vaccination of infants, children, or adults.
- Testing for anti-HBs after vaccination is recommended for the following persons whose subsequent clinical management depends on knowledge of their immune status (Box 7):
  BOX 7 Persons recommended to receive postvaccination serologic testing* following a complete series of HepB vaccination
  • Infants born to hepatitis B surface antigen (HBsAg)–positive mothers or mothers whose HBsAg status remains unknown (e.g., when a parent or person with lawful custody safely surrenders an infant confidentially shortly after birth infants safely surrendered at or shortly after birth)^†^
  • Health care personnel and public safety workers
  • Hemodialysis patients and others who might require outpatient hemodialysis (e.g., predialysis, peritoneal dialysis, and home dialysis)
  • HIV-infected persons
  • Other immunocompromised persons (e.g., hematopoietic stem-cell transplant recipients or persons receiving chemotherapy)
  • Sex partners of HBsAg-positive persons
  * Postvaccination serologic testing for persons other than infants born to HBsAg-positive (or HBsAg-unknown) mothers consists of anti-HBs.
  ^†^ Postvaccination serologic testing for infants born to HBsAg-positive (or HBsAg-unknown) mothers consists of anti-HBs and HBsAg. Persons with anti-HBs <10 mIU/mL after the primary vaccine series should be revaccinated. Infants born to HBsAg-positive mothers or mothers with an unknown HBsAg status should be revaccinated with a single dose of HepB vaccine and receive postvaccination serologic testing 1–2 months later. Infants whose anti-HBs remains <10 mIU/mL following single dose revaccination should receive two additional doses of HepB vaccine, followed by postvaccination serologic testing 1–2 months after the final dose. Based on clinical circumstances or family preference, HBsAg-negative infants with anti-HBs <10 mIU/mL may instead be revaccinated with a second, complete 3-dose series, followed by postvaccination serologic testing performed 1–2 months after the final dose of vaccine. For others with anti-HBs <10 mIU/mL after the primary series, administration of 3 additional HepB vaccine doses on an appropriate schedule, followed by anti-HBs testing 1–2 months after the final dose, is usually more practical than serologic testing after ≥1 dose of vaccine.
  - infants born to HBsAg-positive women and infants born to women whose HBsAg status remains unknown (e.g., infants safely surrendered shortly after birth). Postvaccination serologic testing should consist of testing for anti-HBs and HBsAg;
  - HCP and public safety workers at risk for blood or body fluid exposure;
  - hemodialysis patients (and other persons who might require outpatient hemodialysis), HIV-infected persons, and other immunocompromised persons (e.g., hematopoietic stem-cell transplant recipients or persons receiving chemotherapy), to determine the need for revaccination and the type of follow-up testing; and
  - sex partners of HBsAg-positive persons, to determine the need for revaccination and for other methods of protection against HBV infection.
- Testing should be performed 1–2 months after administration of the final dose of the vaccine series using a method that allows determination of a protective concentration of anti-HBs (≥10 mIU/mL).
  - Persons found to have anti-HBs concentrations of ≥10 mIU/mL after the primary vaccine series are considered to be immune.
  - Immunocompetent persons have long-term protection and do not need further periodic testing to assess anti-HBs levels.
  - Immunocompromised persons might need annual testing to assess anti-HBs concentrations (See Revaccination).
  - Persons found to have anti-HBs concentrations of <10 mIU/mL after the primary vaccine series should be revaccinated. Administration of all doses in the second series, on an appropriate schedule, followed by anti-HBs testing 1–2 months after the final dose, is usually more practical than serologic testing after one or more doses of vaccine (except for when revaccinating infants born to HBsAg-positive mothers).
  - Persons who do not have a protective concentration of anti-HBs after revaccination should be tested for HBsAg.
  - If the HBsAg test result is positive, the person should receive appropriate management, and any household, sexual, or needle-sharing contacts should be identified and vaccinated. Prevaccination testing (consisting of anti-HBc, HBsAg, and anti-HBs) to identify infected persons is recommended for household, sexual, or needle-sharing contacts of HBsAg-positive persons; serologic testing should not be a barrier to vaccination, and the first HepB vaccine dose should be administered immediately after collection of the blood for serologic testing.
  - Persons who test negative for HBsAg should be considered susceptible to HBV infection and should be counseled about precautions to prevent HBV infection and the need to obtain HBIG postexposure prophylaxis for any known or likely exposure to an HBsAg-positive source (*10*).
- Testing HCP with documentation of complete HepB vaccination for anti-HBs upon hire or matriculation (i.e., pre-exposure assessment of prior response to HepB vaccination), followed by one or more additional doses of HepB vaccine for HCP with anti-HBs <10 mIU/mL, helps to ensure that HCP will be protected if they have an exposure to HBV-containing blood or body fluids.
- Anti-HBs levels of ≥10 mIU/mL are generally considered seroprotective; however, different assays have different assay cutoff values based on which reported levels of anti-HBs might vary depending on the assay used. Refer to the package insert of the test for the determination of actual/correct levels of anti-HBs antibodies.

#### Revaccination

- Revaccination (i.e., booster dose, challenge dose, or revaccination with a complete series) is not generally recommended for persons with a normal immune status who were vaccinated as infants, children, adolescents, or adults. Available data do not suggest a maximum number of booster doses. Revaccination when anti-HBs is <10 mIU/mL is recommended for the following persons:
  - **Infants born to HBsAg-positive mothers.** HBsAg-negative infants with anti-HBs <10 mIU/mL should be revaccinated with a single dose of HepB vaccine, and retested 1–2 months later. Infants whose anti-HBs remains <10 mIU/mL following single dose revaccination should receive two additional doses of HepB vaccine on a vaccine schedule to complete the second series, followed by anti-HBs testing 1–2 months later. Alternatively, these infants may be revaccinated with a second 3-dose series and retested (HBsAg and anti-HBs) 1–2 months after the final dose of vaccine.
  - **HCP.** Completely vaccinated HCP with anti-HBs <10 mIU/mL should receive an additional dose of HepB vaccine, followed by anti-HBs testing 1–2 months later. HCP whose anti-HBs remains <10 mIU/mL should complete the second series (usually 6 doses total), followed by repeat anti-HBs testing 1–2 months after the final dose. Alternatively, it might be more practical for very recently vaccinated HCP with anti-HBs <10 mIU/mL to receive the second complete series (usually 6 doses total), followed by anti-HBs testing 1–2 months after the final dose.
  - **Hemodialysis patients.** For hemodialysis patients treated in outpatient centers, the need for booster doses should be assessed by annual anti-HBs testing. A booster dose should be administered when anti-HBs levels decline to <10 mIU/mL. Anti-HBs testing 1–2 months following the booster dose to assess response is not recommended.
  - **Other immunocompromised persons.** For other immunocompromised persons (e.g., HIV-infected persons, hematopoietic stem-cell transplant recipients, and persons receiving chemotherapy), the need for booster doses has not been determined. Annual anti-HBs testing and booster doses should be considered for persons with an ongoing risk for exposure.

#### Interrupted Schedules and Minimum Dosing Intervals

- For all ages, when the HepB vaccine schedule is interrupted, the vaccine series does not need to be restarted. If the series is interrupted after the first dose, the second dose should be administered as soon as possible, and the second and third doses should be separated by an interval of at least 8 weeks. If only the third dose has been delayed, it should be administered as soon as possible. The final dose of vaccine must be administered at least 8 weeks after the second dose and should follow the first dose by at least 16 weeks; the minimum interval between the first and second doses is 4 weeks. Inadequate doses of Hep B vaccine or doses received after a shorter-than-recommended dosing interval should be readministered, using the correct dosage or schedule.
- Vaccine doses administered ≤4 days before the minimum interval or age are considered valid. Because of the unique accelerated schedule for Twinrix, the 4-day guideline does not apply to the first 3 doses of this vaccine when administered on a 0-day, 7-day, 21–30-day, and 12-month schedule (new recommendation).
- In infants, administration of the final dose is not recommended before age 24 weeks.

#### Other Immunization Management Issues

- No differences in immunogenicity have been observed when one or 2 doses of HepB vaccine produced by one manufacturer are followed by doses from a different manufacturer (*8*). Whenever feasible, the same vaccine should be used for the subsequent doses; however, if a different brand is administered, the dose should be considered valid and does not need to be repeated.
- Providers should only accept dated records as evidence of HepB vaccination.
- Although vaccinations should not be postponed if records cannot be found, an attempt to locate missing records should be made by contacting previous health care providers, reviewing state or local immunization information systems, and searching for a personally held record. If records cannot be located within a reasonable time, these persons should be considered susceptible and started on the age-appropriate vaccination schedule. An anti-HBs ≥10 mIU/mL is a serologic correlate of protection only when following a documented, complete series.
- In all settings, vaccination should be initiated even though completion of the series might not be ensured.
- Adverse events occurring after administration of any vaccine should be reported to VAERS. Reports should be submitted to VAERS online, by facsimile, or by mail. More information about VAERS is available by calling 1-800-822-7967 (toll-free) or online at https://vaers.hhs.gov.

## Future Directions

ACIP and CDC will review these recommendations as new epidemiology or other information related to HepB vaccines (including licensure of additional HepB-containing vaccines), HepB vaccine adverse events, and the experience gained in the implementation of these recommendations becomes available. Revised recommendations will be developed as needed.

## Footnotes

* A list of the members appears on page XX.

† Code of Federal Regulations. 21 CFR §600.80. Revised April 1, 2010. Available at https://www.accessdata.fda.gov/scripts/cdrh/cfdocs/cfcfr/CFRSearch.cfm? fr=600.80.
